# Supplementary material for: Light-driven transformable optical agent with adaptive functions for boosting cancer surgery outcomes
Source: Nat Commun. 2018 May 10;9:1848. doi: 10.1038/s41467-018-04222-8 (PMC5945617; doi:10.1038/s41467-018-04222-8)
Supplement: Supplementary file 1 — Supplementary Information [file 41467_2018_4222_MOESM1_ESM.pdf]

Supplementary Information

Light-driven transformable optical agent with adaptive functions for boosting cancer surgery outcomes

Qi *et al.*

Supplementary Methods

General methods. All the chemicals and reagents were purchased from Sigma-Aldrich unless otherwise specified, and were used as received. 1,2-Distearoyl-*sn*-glycero-3-phosphoethanolamine-*N*-[(polyethylene glycol)-2000]-maleimide (DSPE-PEG_{2000}-maleimide) was obtained from Laysan Bio, Inc. (Arab, AL). Cell culture RPMI 1640 medium and fetal bovine serum (FBS) were provided by Thermo Fisher Scientific Inc. (Waltham, MA, USA). The solvents for chemical reactions were distilled before use. The nuclear magnetic resonance (NMR) spectra were recorded on a Bruker AV 400 spectrometer. High-resolution mass spectra (HRMS) were measured with a GCT premier CAB048 mass spectrometer in matrix assisted laser desorption ionization-time of flight (MALDI-TOF) mode. The geometry optimization was calculated at the level of B3LYP/6-31G* using density functional theory (DFT) method with the Gaussian 09 program package. The UV-vis absorption spectra were performed using a Milton Roy Spectronic 3000 Array spectrophotometer. Steady-state photoluninescence (PL) spectra were conducted on a Horiba Fluorolog-3 spectrofluorometer. The photoluminescence quantum yield (QY) was measured using a Hamamatsu absolute PL quantum yield spectrometer C11347 Quantaurus-QY. Transient PL at room temperature was measured using an Edinburgh FLSP980 fluorescence spectrophotometer. The X-ray diffraction (XRD) profiles were obtained using a Bruker D8 Discover reflector. Transmission electron microscopy (TEM) images were acquired from a JEM-2010F transmission electron microscope with an accelerating voltage of 200 kV. Dynamic light scattering (DLS) was measured on a 90 plus particle size analyzer.

Peptide synthesis. The peptide with the sequence of CYSAYPDSVPMMS (YSA in short) was synthesized through standard 9-fluorenylmethoxycarbonyl (Fmoc) solid-phase peptide synthesis (SPPS) using 2-chlorotrityl chloride resin and the corresponding *N*-Fmoc protected amino acids

with side chains properly protected. The growth of the peptide chain was according to the established Fmoc SPPS protocol. After the last coupling step, excessive reagents were removed by a single *N*,*N'*-dimethylformamide (DMF) washing for 5 min (5 mL per gram of resin), followed by 5 steps of washing with dichloromethane (DCM) for 1 min (5 mL per gram of resin). The peptide derivative was cleaved using 95% of trifluoroacetic acid (TFA) with 2.5% of trimethylsilane (TMS) and 2.5% of H_2O for 30 min. Then, 20 mL per gram of resin of ice-cold diethylether was added to the cleavage reagent. After that, the obtained product was purified by HPLC. The peptide was obtained in 78% yield. HRMS, *m/z* calcd. for $C_{62}H_{91}N_{13}O_{21}S_3^+$: 1449.5615; found 1450.5624 $(M + H)^+$.

Calculation of YSA peptides modified on each NP. The stock solution (9.0 mL) of RClosed NPs without YSA conjugation was first freeze-dried for 2 days to yield the fine powder (2.8 mg). Considering the fact that the NPs are stable in water, the density of the NP suspension could be estimated as ~1 g cm^{-3}. As the average hydrodynamic diameter of the RClosed NPs measured by DLS is ~65 nm, the NP concentration in stock solution could be calculated from the following equation:

$$\text{Total number of RClosed NPs in 9.0 mL of stock solution}$$
$$= \frac{\text{Total volume of NPs}}{\text{Average volume of each NP}} = \frac{\dfrac{2.8\times10^{-3}\,\text{g}}{1\,\text{g/mL}}}{\dfrac{4}{3}\pi\times(32.5\times10^{-7})^3\,\text{mL}} = 1.95\times10^{13} \tag{1}$$

$$\text{NP concentration} = \frac{\dfrac{1.95\times10^{13}}{6.02\times10^{23}\,\text{mol}^{-1}}}{9.0\times10^{-3}\,\text{L}} = 3.60\text{ nM} \tag{2}$$

To conjugate CYSAYPDSVPMMS peptides on the RClosed NPs, 0.2 μmol of peptides were fed to react with NPs. The free peptides that were not conjugated on the NPs were then removed by ultrafiltration. The amount of free peptides was determined by HPLC, which

revealed that 0.08 μmol peptides were not conjugated on the NPs. As a result, the average number of CYSAYPDSVPMMS peptides modified on each NP is:

$$\frac{0.12\times10^{-6}\,\text{mol}}{3.6\times10^{-9}\,\text{mol/L}\times9.0\times10^{-3}\,\text{L}}=3704 \tag{3}$$

Western blot analysis. The EphA2 protein levels in 4T1 cancer cells and hepatic L02 normal cells were analyzed by Western blot. Briefly, the cells were firstly collected and lysed. Total proteins were then extracted and loaded in the wells of 10% sodium dodecyl sulfate-polyacrylamide gel electrophoresis (SDS-PAGE). After the separation by gel electrophoresis, the proteins were transferred to PVDF membranes (Millipore, Billerica, MA, USA). The membrane was probed with primary antibodies after being blocked by 5% non-fat milk (Bio-Rad) overnight at 4 ^{o}C. Primary antibodies included rabbit monoclonal anti-mouse, rat, human GAPDH antibody (catalog no: ab201822, clone: EPR 16891, Abcam)[1], and rabbit polyclonal anti-mouse, hamster, human Eph receptor A2 antibody (catalog no: ab5387, Abcam)[2]. After washing with TBST, membranes were incubated with corresponding HRP-conjugated secondary antibody for 1 h at room temperature. The membrane was washed 3 times by TBST, then protein bands were visualized on the Tanon-5200 Chemiluminescent imaging System (Tanon Science and Technology). Uncropped original scans of blots are shown in Supplementary Fig. 30.

***In vitro* cell imaging.** The 4T1 cancer cells were cultured in confocal imaging chambers at 37 ^{o}C. On the day of experiment, the medium in the chambers were removed and the ROpen-YSA NPs as well as ROpen NPs (both at 8 μM based on ROpen-DTE-TPECM) in FBS-free cell culture medium were subsequently added to the chambers, respectively. After incubation at 37 ^{o}C for 4 h, both NP-treated cells were washed three times with 1 × PBS buffer, followed by imaging with confocal laser scanning microscope (CLSM, Leica TSC SP8, Germany) upon excitation at 405 nm with a 505 nm longpass barrier filter. Alternatively, the RClosed-YSA NPs (8 μM based on

RClosed-DTE-TPECM) in FBS-free cell culture medium were added to the chambers containing 4T1 cancer cells, and incubated for 4 h. After that, the RClosed-YSA NP-incubated 4T1 cells were washed with 1 × PBS and irradiated with 610 nm red light (0.3 W cm^{-2}) for 5 min. The RClosed-YSA NP-incubated cells before and after red light exposure were imaged by CLSM.

Intracellular ROS detection. The 4T1 cancer cells were incubated with RClosed-YSA NPs (8 μM based on RClosed-DTE-TPECM) for 4 h, followed by 610 nm red light (0.3 W cm^{-2}) irradiation for 5 min in order to convert RClosed-YSA NPs into ROpen-YSA NPs. Alternatively, the NP-treated cells were co-treated with NaN_3 (10 mM). ROS indicator DCF-DA (10 μM) was then loaded into the cells. After 5 min incubation, the cells were washed with 1 × PBS and exposed to white light (0.25 W cm^{-2}) for 2 min. Afterward, the cells were washed with 1 × PBS and immediately imaged by CLSM. For DCF detection, excitation at 488 nm and signal collection at 520 ± 10 nm were adopted. In addition, the ROS level inside the NP-treated cells without exposure to white light was also examined using DCF-DA as the indicator, following the same experiment procedures.

Cytotoxicity study. The 4T1 cancer cells were seeded in a 96 well plate with a density of 5,000 cells per well and allowed to adhere for 24 h prior to the 3-(4,5-dimethylthiazol-2-yl)-2,5-diphenyl tetrazolium bromide (MTT) assay. The cells were then incubated with a series of doses of RClosed-YSA NPs and RClosed NPs for 4 h, respectively, followed by washing with 1 × PBS and exposure to 610 nm red light (0.3 W cm^{-2}) irradiation for 5 min in order to convert ring-closing NPs into ring-opening NPs. After that, both the NP-treated cells were exposed to white light (0.25 W cm^{-2}) for 4 min. Alternatively, the NP-treated cells were kept in dark without white light irradiation. After 24 h of culture in fresh medium, 100 μL of freshly prepared MTT solution (0.5 mg mL^{-1}) in culture medium was added into each well. After incubation for 3 h, the

supernatant was discarded and the precipitate was dissolved in 100 μL of DMSO with gentle shaking. The absorbance of MTT at 570 nm was measured by the microplate reader (GENios Tecan). Cell viability was expressed by the ratio of the absorbance of the cells incubated with the NPs to that of the cells incubated with culture medium only.

Radiolabelling of RClosed-YSA NPs with iodine-125 (^{125}I). The RClosed-YSA NPs were labelled with ^{125}I on the phenol group of the tyrosine (Y) residue in YSA peptide using chloramine-T strategy. Briefly, to an aqueous solution of RClosed-YSA NPs (900 μL, 800 μM based on RClosed-DTE-TPECM), Na^{125}I in PBS buffer (10 mM) was added, followed by reaction at room temperature for 30 min. The reaction was subsequently quenched by adding 100 μL of sodium peroxodisulfate in PBS (10 mM) buffer. The unreacted ^{125}I and other chemicals were removed by centrifugation in an Amicon Ultra-4 device with an ultrafiltration membrane (molecule weight cutoff 100,000 Da) at a speed of 4000 rpm. The labelling rate and radiochemical purity of the NPs were examined by radioactive thin-layer chromatography (TLC) scanner with mobile phase containing 90% ethanol and 10% deionized water.

Pharmacokinetics and biodistribution studies. Pharmacokinetics study was carried out according to the literature[3]. Male and female Sprague–Dawley rats were randomly selected for the following experiment. The rats were anaesthetized, and then a midline incision of the neck region was made for each rat. The jugular vein and the contralateral carotid artery were cannulated with PE-50 tubing and flushed using heparin-containing saline solution. The ^{125}I-labelled RClosed-YSA NPs (250 μL, 3.2 mM based on RClosed-DTE-TPECM) were intravenously injected into the rats *via* the jugular vein (n = 6 with 3 male and 3 female rats). At different time points post-injection, 0.2 mL of blood samples were retrieved from the carotid artery and counted for ^{125}I radioactivity with a gamma counter (2470 WIZARD, PerkinElmer,

USA). The pharmacokinetic parameters were shown in Supplementary Table 1, which were analyzed according to a non-compartment model by DAS 3.0.

For the biodistribution study, the xenograft 4T1 tumour-bearing mice were randomly selected for the following treatments. The tumour-bearing mice were intravenously injected with ^{125}I-labelled RClosed-YSA NPs (100 μL, 800 μM based on RClosed-DTE-TPECM) through the tail vein. At designated time intervals, the mice were sacrificed with 6 mice for each time point. The blood was collected as well as a variety of tissues including the skin, muscle, intestine (emptied), heart, lung, liver, spleen, stomach (emptied), bone and tumour were harvested. The tissues were weighted and the amounts of radioactivity in the biological samples were measured by the gamma counter. The results were defined as the percentage of the injected dose per gram of tissue (% ID g^{-1}).

Synthesis and characterization.

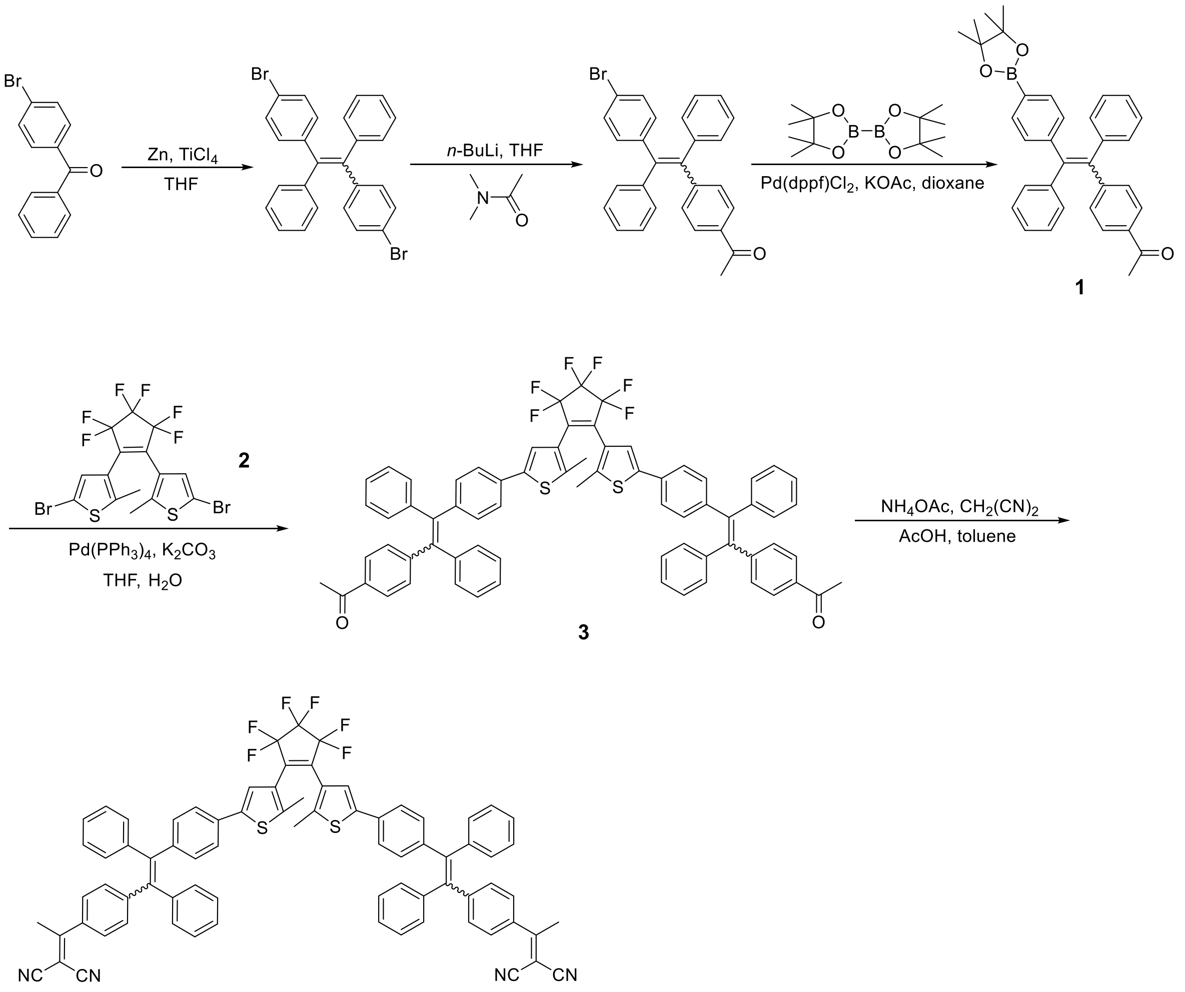

ROpen-DTE-TPECM

Supplementary Figure 1. Synthesis route to compound ROpen-DTE-TPECM.

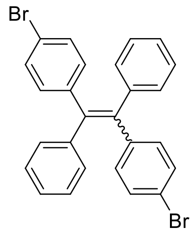

1,2-Bis(4-bromophenyl)-1,2-diphenylethene

(4-Bromophenyl)(phenyl)methanone (7.83 g, 30 mmol) and zinc powder (7.8 g, 120 mmol) were added into a 250 mL two-necked round-bottom flask. The flask was vacuumed and purged with dry nitrogen three times. Then anhydrous THF (150 mL) were added, and the mixture was cooled with ice-water to 0 ^{o}C. $TiCl_4$ (9.9 mL, 90 mmol) was added dropwise, and the mixture was heated to reflux and stirred for 12 h. After cooling down to room temperature, aqueous HCl (1 M) was added, the mixture was stirred for 2 h and extracted with dichloromethane three times. The organic phase was combined and washed with water, then dried with $MgSO_4$. After removal of the solvent under reduced pressure, the crude product was purified by column chromatography on silica gel using dichloromethane/hexane (v/v 1:8) as the eluent to result in 1,2-bis(4-bromophenyl)-1,2-diphenylethene as a white solid (79% yield). ^{1}H NMR (400 MHz, $CDCl_3$): δ 7.26-7.19 (m, 4H), 7.16-7.07 (m, 6H), 7.02-6.95 (m, 4H), 6.91-6.84 (m, 4H). HRMS (m/z): $[M]^+$ calcd. for $C_{26}H_{18}Br_2$, 489.9775; found, 489.9792.

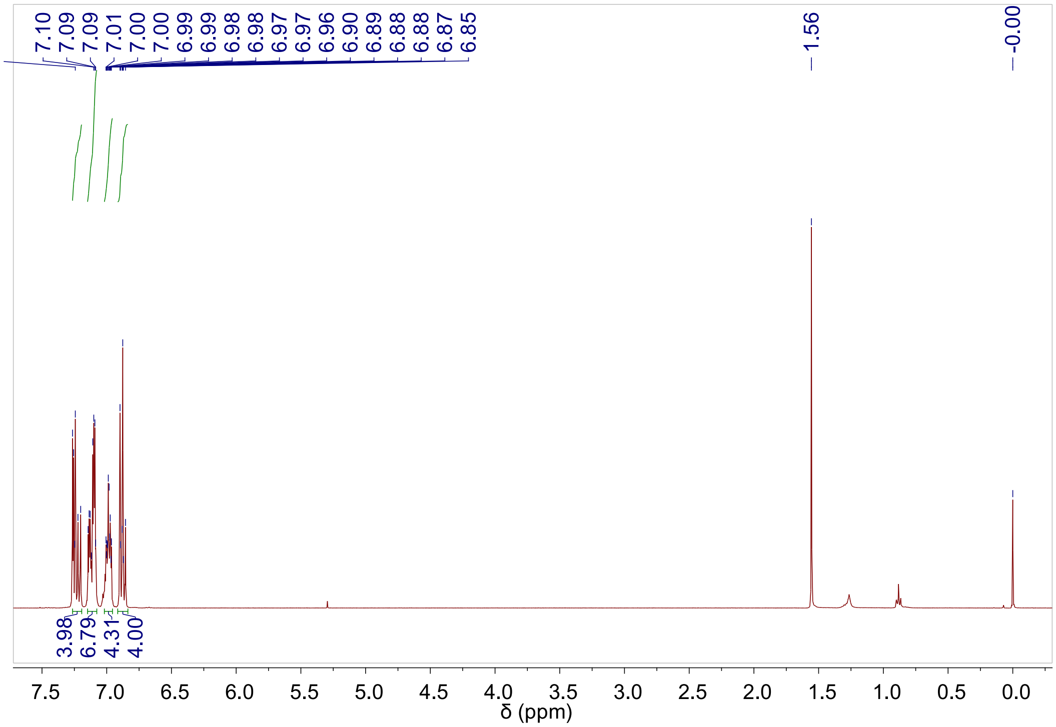

Supplementary Figure 2. ^{1}H NMR spectrum of 1,2-bis(4-bromophenyl)-1,2-diphenylethene in $CDCl_3$ at 298 K.

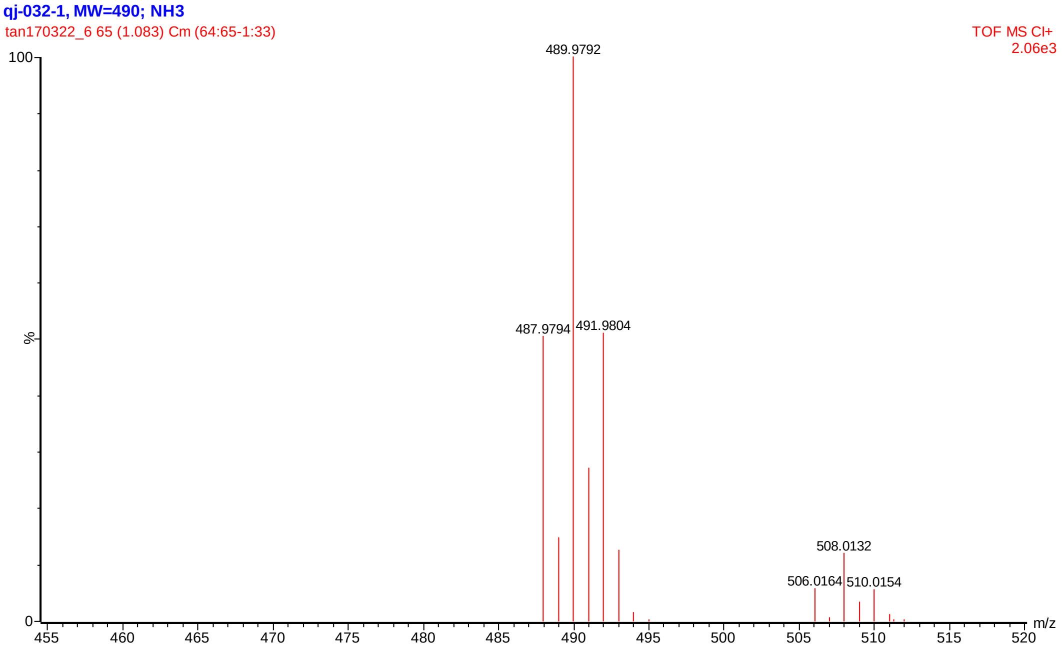

Supplementary Figure 3. HRMS of 1,2-bis(4-bromophenyl)-1,2-diphenylethene.

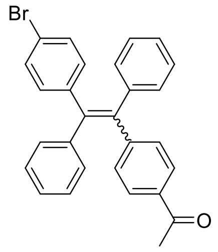

1-(4-(2-(4-Bromophenyl)-1,2-diphenylvinyl)phenyl)ethan-1-one

1,2-Bis(4-bromophenyl)-1,2-diphenylethene (2.45 g, 5 mmol) was added into a 100 mL two-necked round-bottom flask. The flask was vacuumed and purged with dry nitrogen three times. Then anhydrous THF (40 mL) were added, and the mixture was cooled down with the mixture of dry ice-acetone to -78 ^{o}C, and maintained for 15 min, followed by the addition of *n*-butyllithium (^{n}BuLi, 2.5 M hexane solution, 2.4 mL, 6 mmol). The reaction mixture was stirred at -78 ^{o}C for 1 h, and dry *N*,*N*-dimethylacetamide (1 mL) was added. Afterward, the mixture was slowly warmed to room temperature and stirred overnight. The reaction was quenched by the addition of saturated ammonium chloride aqueous solution, and the mixture was extracted with dichloromethane three times. The organic phase was combined, and dried with $MgSO_4$. After removal of the solvent under reduced pressure, the crude product was purified by column chromatography on silica gel using dichloromethane/hexane (v/v 1:1) as the eluent to afford 1-(4-(2-(4-bromophenyl)-1,2-diphenylvinyl)phenyl)ethan-1-one as a light yellow solid (62% yield). ^{1}H NMR (400 MHz, $CDCl_3$): δ 7.73-7.68 (m, 4H), 7.16-7.08 (m, 10H), 7.02-6.97 (m, 4H), 2.53 (s, 3H). HRMS (m/z): $[M]^+$ calcd. for $C_{28}H_{21}OBr$, 452.0776; found, 452.0756.

1-one.

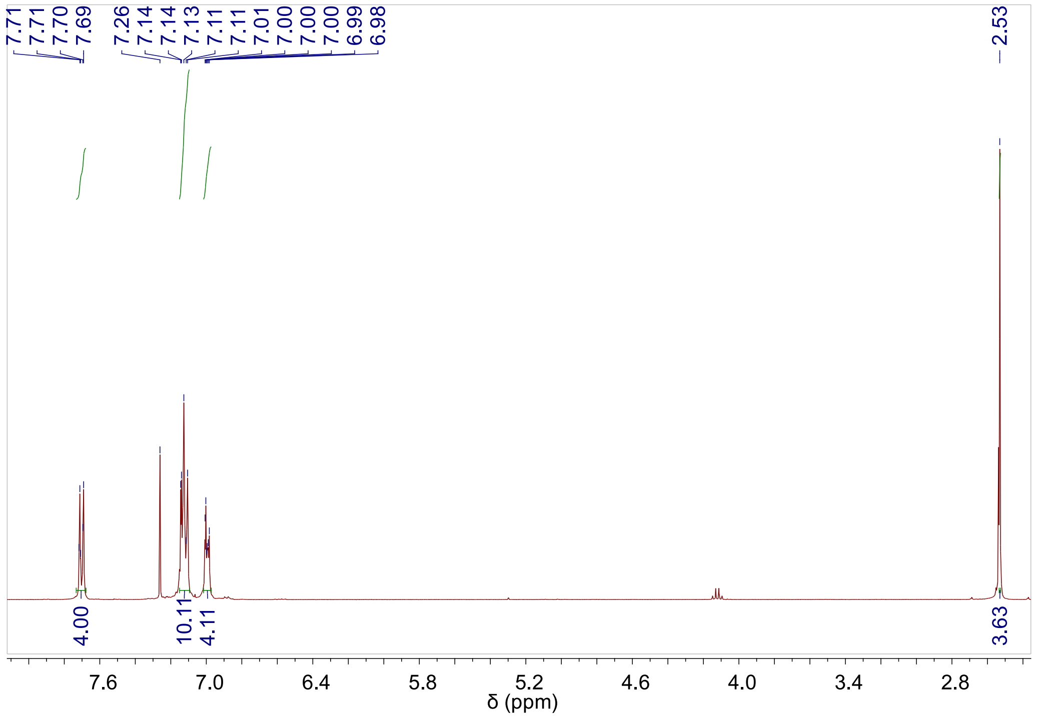

Supplementary Figure 4. ^{1}H NMR spectrum of 1-(4-(2-(4-bromophenyl)-1,2-diphenylvinyl)phenyl)ethan-1-one in $CDCl_3$ at 298 K.

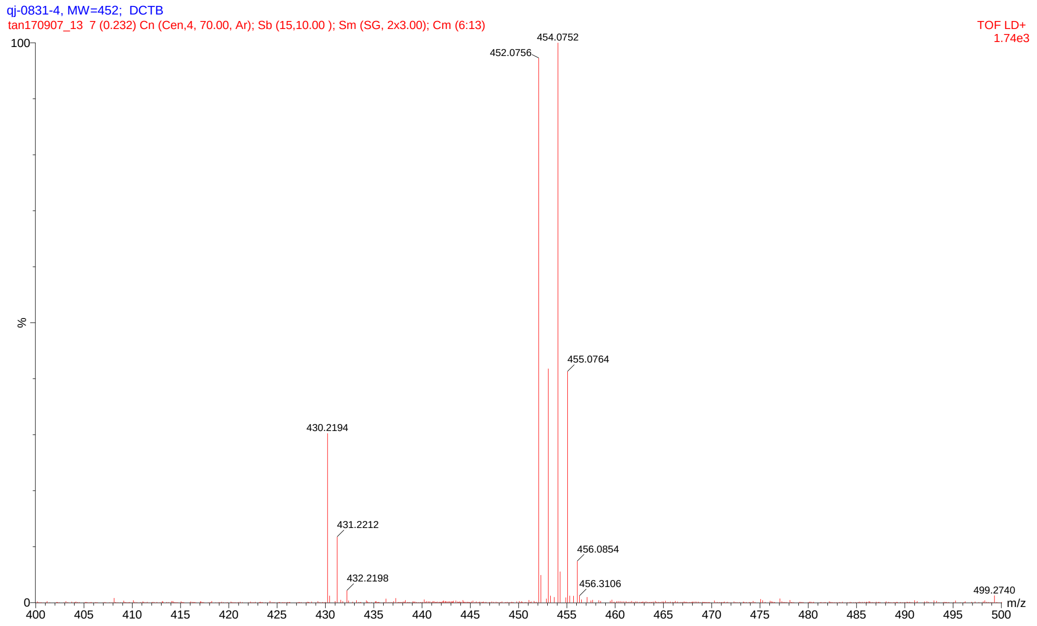

Supplementary Figure 5. HRMS of 1-(4-(2-(4-bromophenyl)-1,2-diphenylvinyl)phenyl)ethan-1-one.

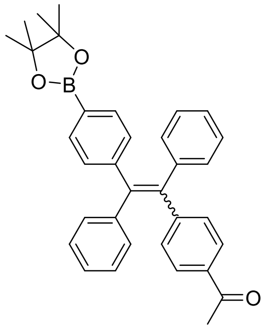

1-(4-(1,2-Diphenyl-2-(4-(4,4,5,5-tetramethyl-1,3,2-dioxaborolan-2-yl)phenyl)vinyl)phenyl)ethan-1-one (**1**)

1-(4-(2-(4-Bromophenyl)-1,2-diphenylvinyl)phenyl)ethan-1-one (1.36 g, 3 mmol), bis(pinacolato)diboron (1.14 g, 4.5 mmol), Pd(dppf)Cl_2 (110 mg, 0.15 mmol) and KOAc (0.88 g, 9 mmol) were added into a 100 mL two-necked round-bottom flask. The flask was vacuumed and purged with dry nitrogen three times. Then anhydrous 1,4-dioxane (40 mL) was added, and the mixture was heated to reflux and stirred for 24 h. The reaction mixture was cooled down to room temperature, and water was added, followed by extraction with dichloromethane three times. The organic phase was combined, and dried with $MgSO_4$. After removal of the solvent under reduced pressure, the crude product was purified by column chromatography on silica gel using dichloromethane/hexane (v/v 2:1) as the eluent to afford 1-(4-(1,2-diphenyl-2-(4-(4,4,5,5-tetramethyl-1,3,2-dioxaborolan-2-yl)phenyl)vinyl)phenyl)ethan-1-one (**1**) as a light yellow solid (91% yield). ^{1}H NMR (400 MHz, $CDCl_3$): δ 7.69 (d, 2H), 7.5 (d, 2H), 7.13-7.07 (m, 8H), 7.05-6.97 (m, 6H), 2.53 (d, 3H), 1.32 (d, 12H). HRMS (m/z): $[M]^+$ calcd. for $C_{34}H_{33}OB_3$, 500.2523; found, 500.2556.

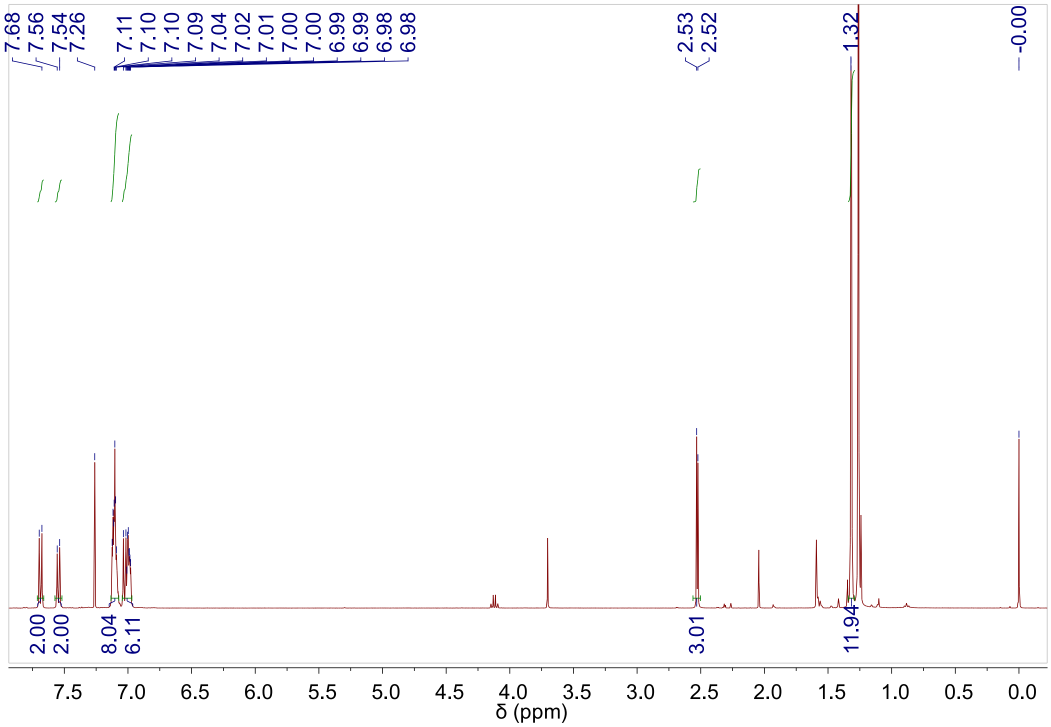

Supplementary Figure 6. ^{1}H NMR spectrum of compound **1** in $CDCl_3$ at 298 K.

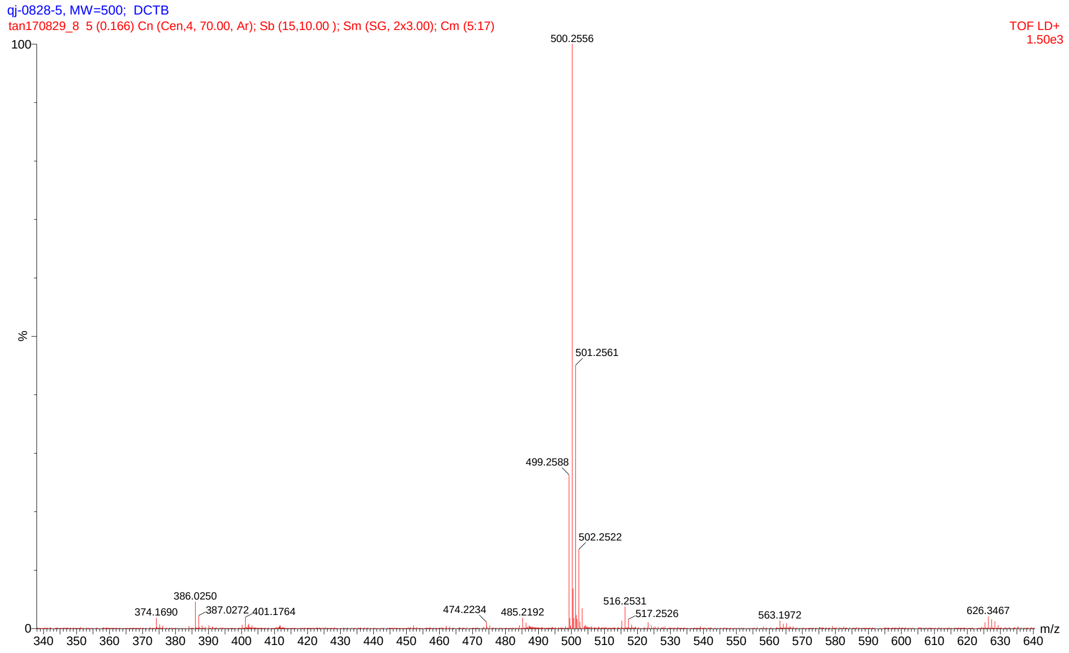

Supplementary Figure 7. HRMS of compound **1**.

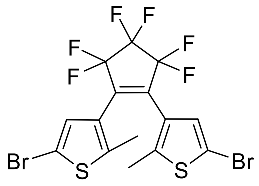

3,3'-(Perfluorocyclopent-1-ene-1,2-diyl)bis(5-bromo-2-methylthiophene) (**2**)

It was synthesized as the procedures reported in the literatures[4-6]. The product was obtained as a white solid. ^{1}H NMR (400 MHz, $CDCl_3$): δ 7.01 (s, 2H), 1.88 (s, 6H). ^{13}C NMR (100 MHz, $CDCl_3$): δ 143.34, 135.64, 129.16, 125.19, 115.82, 113.50, 110.07, 14.39. ^{19}F NMR (376 MHz, $CDCl_3$): δ -110.30 (t, 4F), -131.91 (m, 2F). HRMS (m/z): $[M]^+$ calcd. for $C_{15}H_8S_2F_6Br_2$, 525.8318; found, 525.8333.

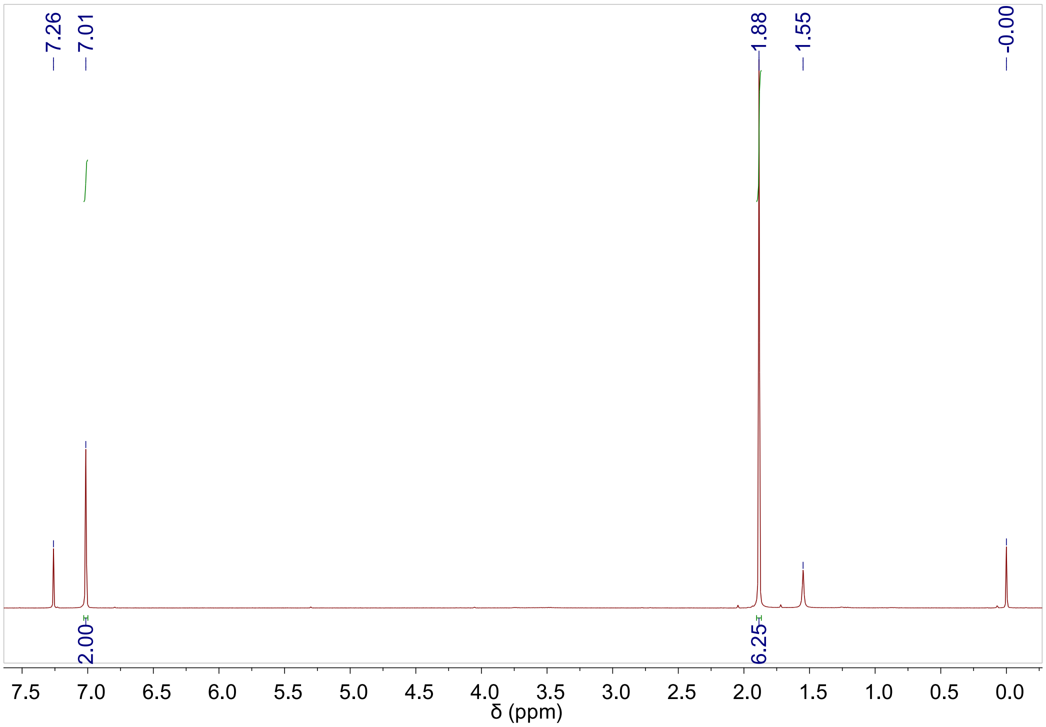

Supplementary Figure 8. ^{1}H NMR spectrum of compound **2** in $CDCl_3$ at 298 K.

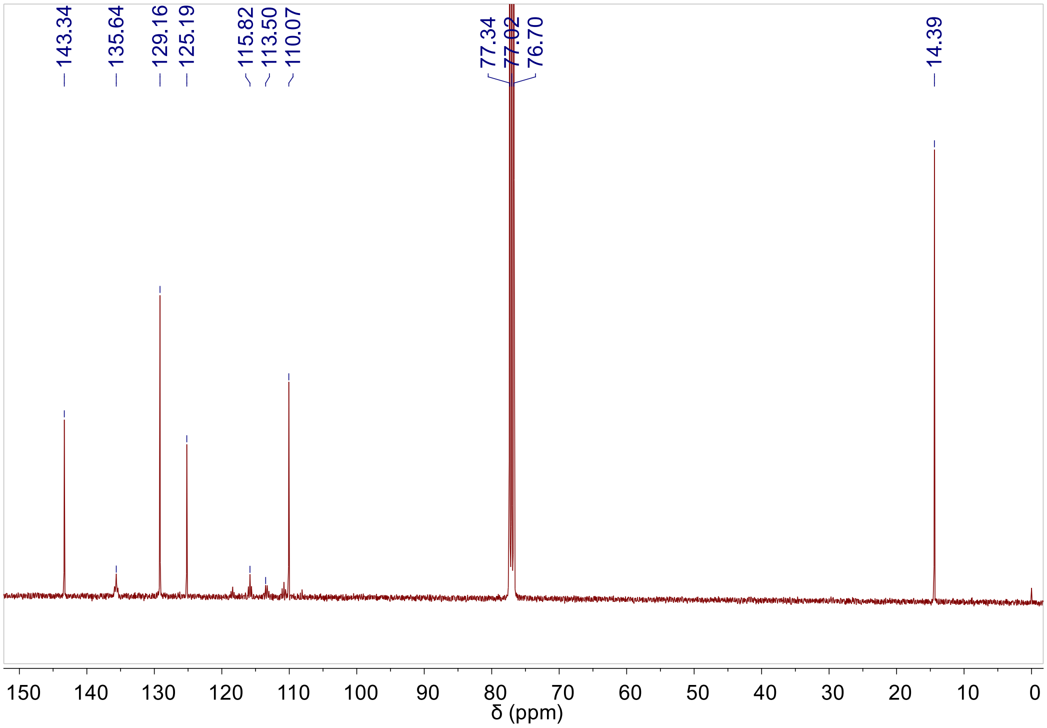

Supplementary Figure 9. ^{13}C NMR spectrum of compound **2** in $CDCl_3$ at 298 K.

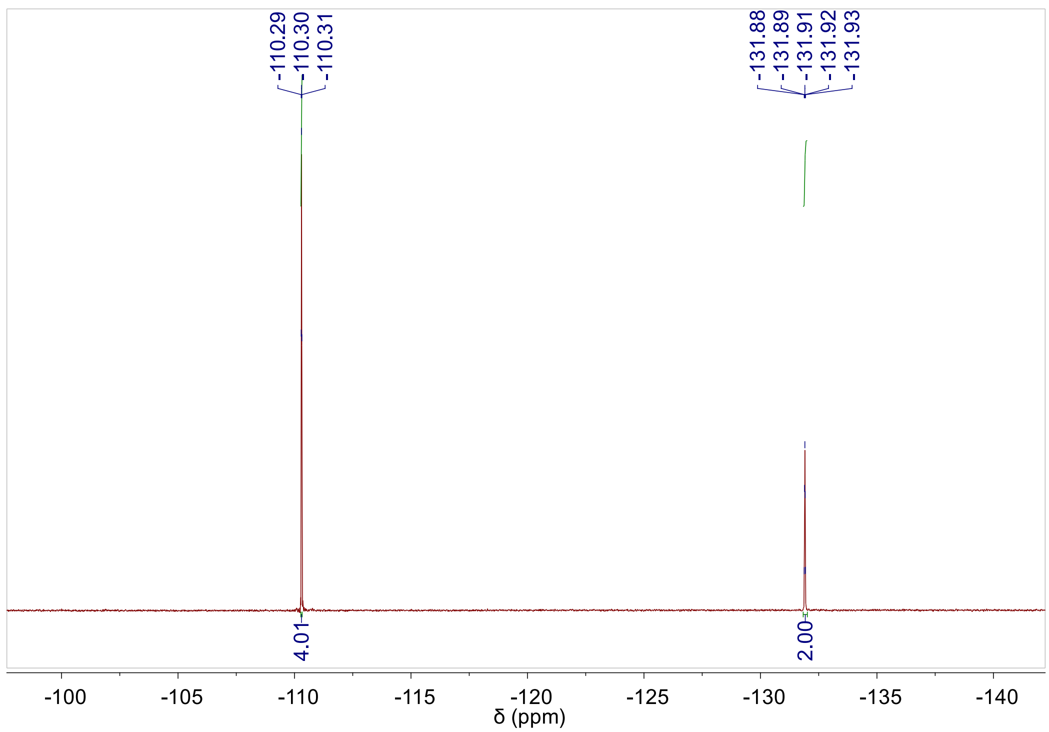

Supplementary Figure 10. ^{19}F NMR spectrum of compound **2** in $CDCl_3$ at 298 K.

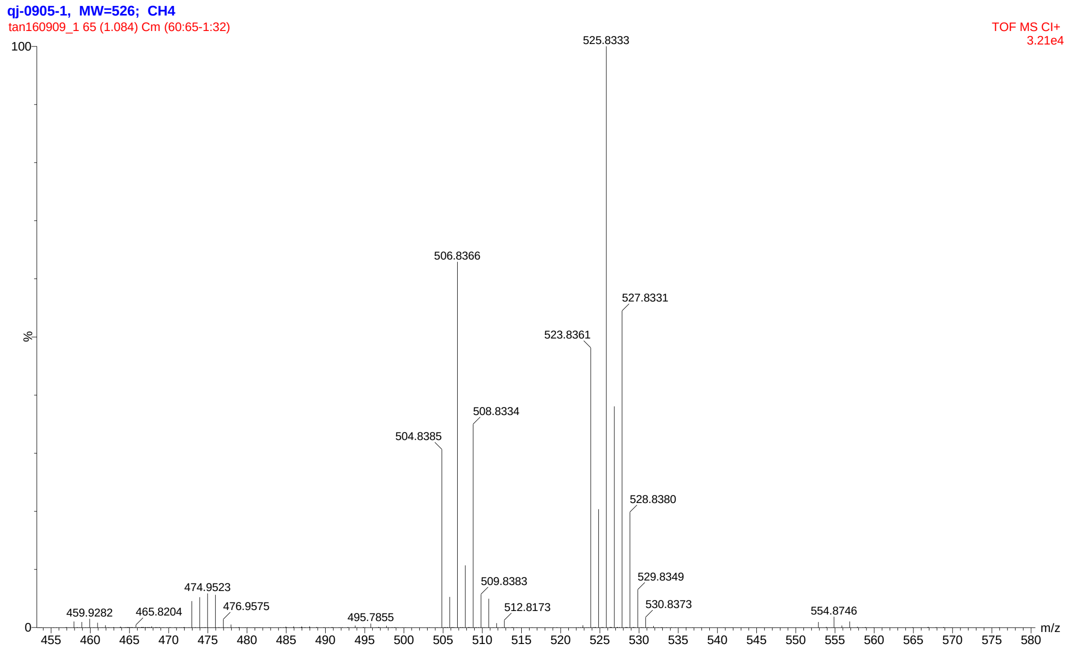

Supplementary Figure 11. HRMS of compound **2**.

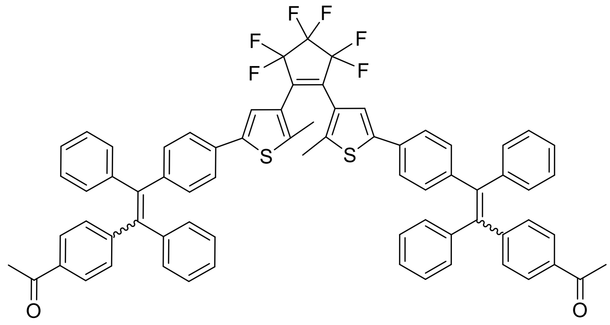

1,1'-(((((Perfluorocyclopent-1-ene-1,2-diyl)bis(5-methylthiophene-4,2-diyl))bis(4,1-phenylene))bis(1,2-diphenylethene-2,1-diyl))bis(4,1-phenylene))bis(ethan-1-one) (**3**)

Compound **1** (1.0 g, 2 mmol), compound **2** (0.47 g, 0.9 mmol) and $Pd(PPh_3)_4$ (0.12 g, 0.1 mmol) were added into a 100 mL two-necked round-bottom flask. The flask was vacuumed and purged with dry nitrogen three times. Then THF (30 mL) and aqueous K_2CO_3 solution (2 M, 10 mL) was added, and the mixture was heated to reflux, and stirred for 24 h. Water was added and the mixture was extracted with dichloromethane three times. The organic phase was combined, and dried with $MgSO_4$. After removal of the solvent under reduced pressure, the crude product was purified by column chromatography on silica gel using dichloromethane/hexane (v/v 1:1) as the eluent to afford 1,1'-(((((perfluorocyclopent-1-ene-1,2-diyl)bis(5-methylthiophene-4,2-diyl))bis(4,1-phenylene))bis(1,2-diphenylethene-2,1-diyl))bis(4,1-phenylene))bis(ethan-1-one) (**3**) as a yellow solid (76% yield). ^{1}H NMR (400 MHz, $CDCl_3$): δ 7.78-7.68 (m, 4H), 7.39-7.30 (d, 4H), 7.24-7.00 (m, 30H), 2.52 (d, 6H), 1.95 (d, 6H). HRMS (m/z): $[M]^+$ calcd. for $C_{71}H_{50}O_2S_2F_6$, 1112.3156; found, 1112.3152.

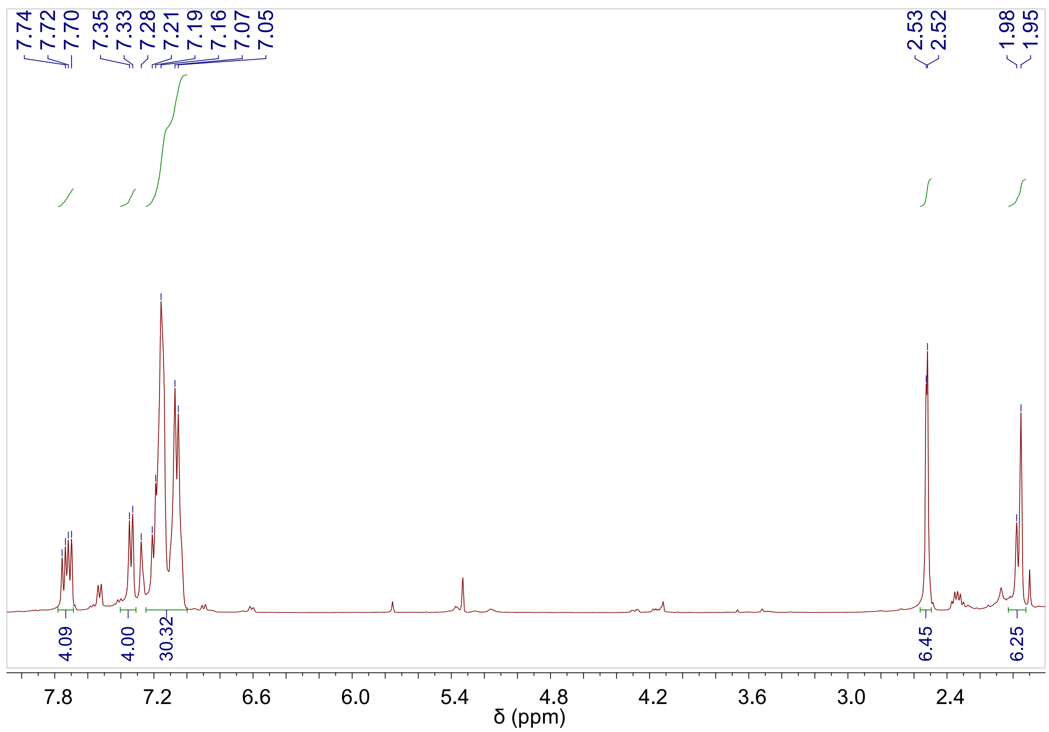

Supplementary Figure 12. ^{1}H NMR spectrum of compound **3** in $CDCl_3$ at 298 K.

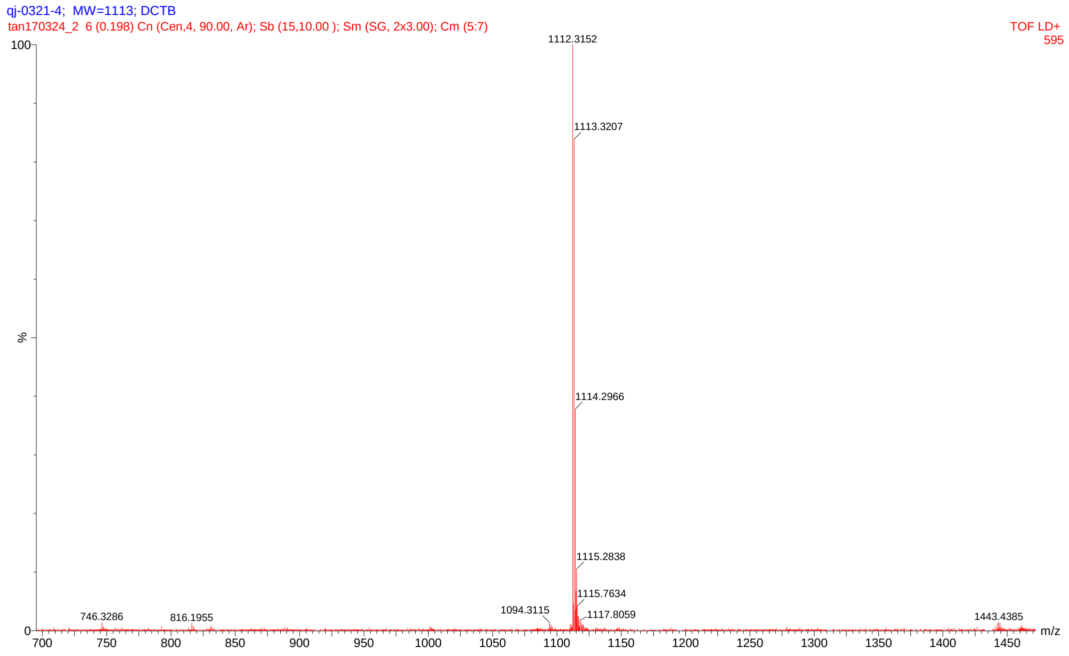

Supplementary Figure 13. HRMS of compound **3**.

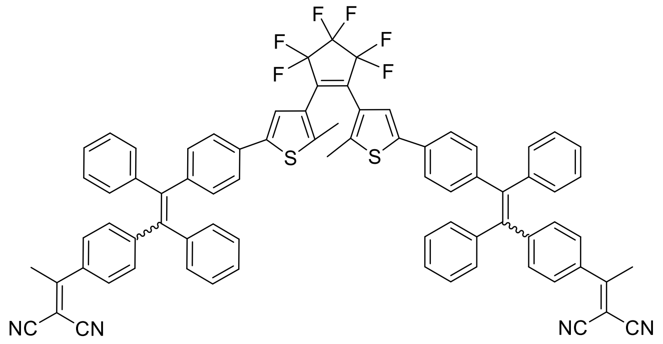

2,2'-((((((Perfluorocyclopent-1-ene-1,2-diyl)bis(5-methylthiophene-4,2-diyl))bis(4,1-phenylene))bis(1,2-diphenylethene-2,1-diyl))bis(4,1-phenylene))bis(ethan-1-yl-1-ylidene))dimalononitrile (**ROpen-DTE-TPECM**)

Compound **3** (0.56 g, 0.5 mmol), malononitrile (0.1 g, 1.5 mmol) and ammonium acetate (0.12 g, 1.5 mmol) were added into a 100 mL two-necked round-bottom flask. The flask was vacuumed and purged with dry nitrogen three times. Then anhydrous toluene (40 mL) and acetic acid (1 mL) were added, and the mixture was heated to reflux and stirred for 4 h. After cooling down to room temperature, water was added, and the mixture was extracted with dichloromethane. The organic phase was combined and dried with $MgSO_4$. After removal of the solvent under reduced pressure, the crude product was purified by column chromatography on silica gel using dichloromethane/hexane (v/v 2:1) as the eluent to afford 2,2'-((((((perfluorocyclopent-1-ene-1,2-diyl)bis(5-methylthiophene-4,2-diyl))bis(4,1-phenylene))bis(1,2-diphenylethene-2,1-diyl))bis(4,1-phenylene))bis(ethan-1-yl-1-ylidene))dimalononitrile (**ROpen-DTE-TPECM**) as a yellow solid (78% yield). ^{1}H NMR (400 MHz, CD_2Cl_2): δ 7.44-7.33 (m, 8H), 7.31-7.26 (m, 2H), 7.25-7.15 (m, 16H), 7.14-7.04 (m, 12H), 2.60 (d, 6H), 1.98 (d, 6H). HRMS (m/z): $[M]^+$ calcd. for $C_{77}H_{50}N_4S_2F_6$, 1208.3381; found, 1208.3357.

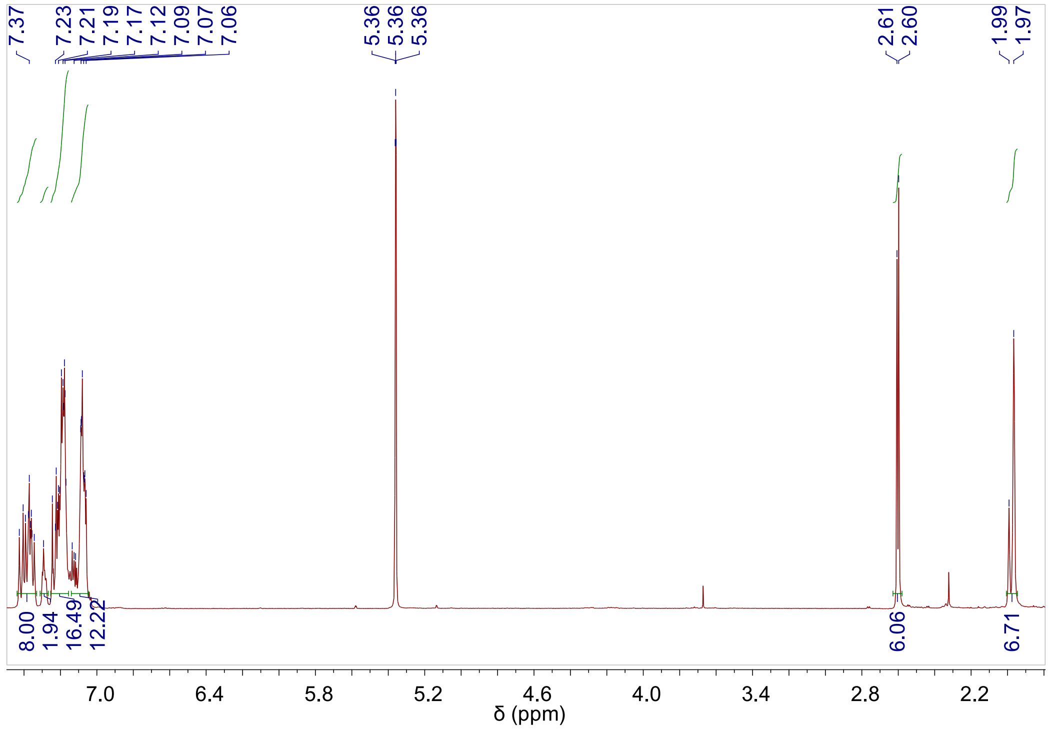

Supplementary Figure 14. ^{1}H NMR spectrum of compound **ROpen-DTE-TPECM** in CD_2Cl_2 at 298 K.

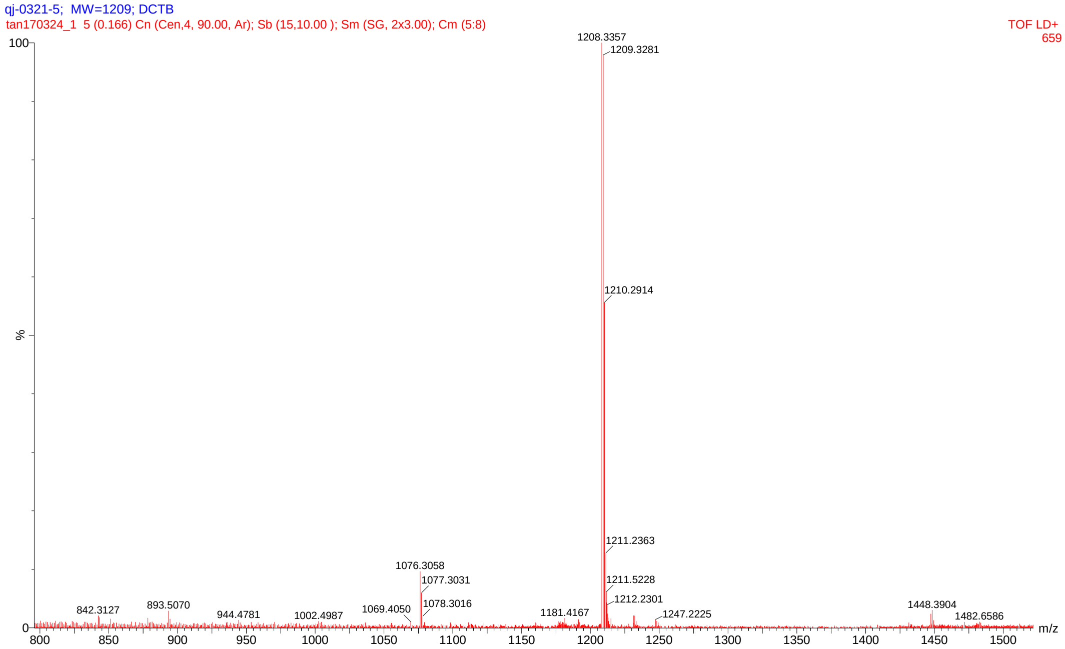

Supplementary Figure 15. HRMS of compound **ROpen-DTE-TPECM**.

RClosed-DTE-TPECM. ^{1}H NMR (400 MHz, CD_2Cl_2): δ 7.45-7.33 (m, 8H), 7.32-7.26 (m, 2H), 7.26-7.15 (m, 16H), 7.14-7.03 (m, 12H), 2.60 (d, 6H), 1.97 (d, 6H). HRMS (m/z): $[M]^+$ calcd. for $C_{77}H_{50}N_4S_2F_6$, 1208.3381; found, 1208.3350.

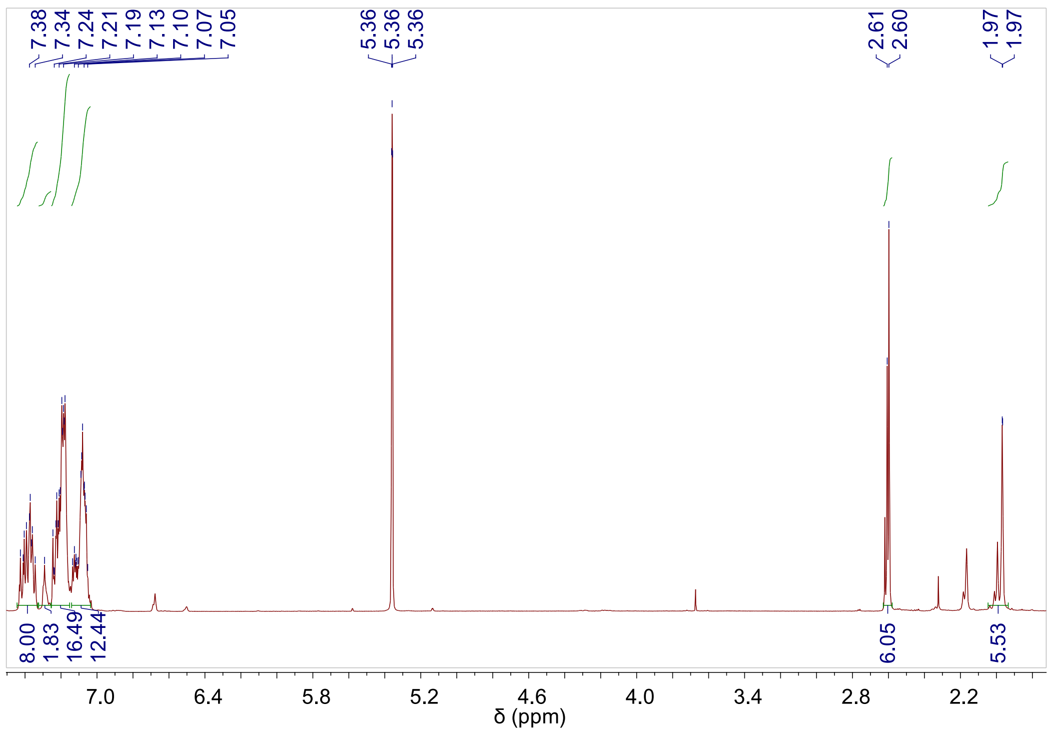

Supplementary Figure 16. ^{1}H NMR spectrum of compound **RClosed-DTE-TPECM** in CD_2Cl_2 at 298 K.

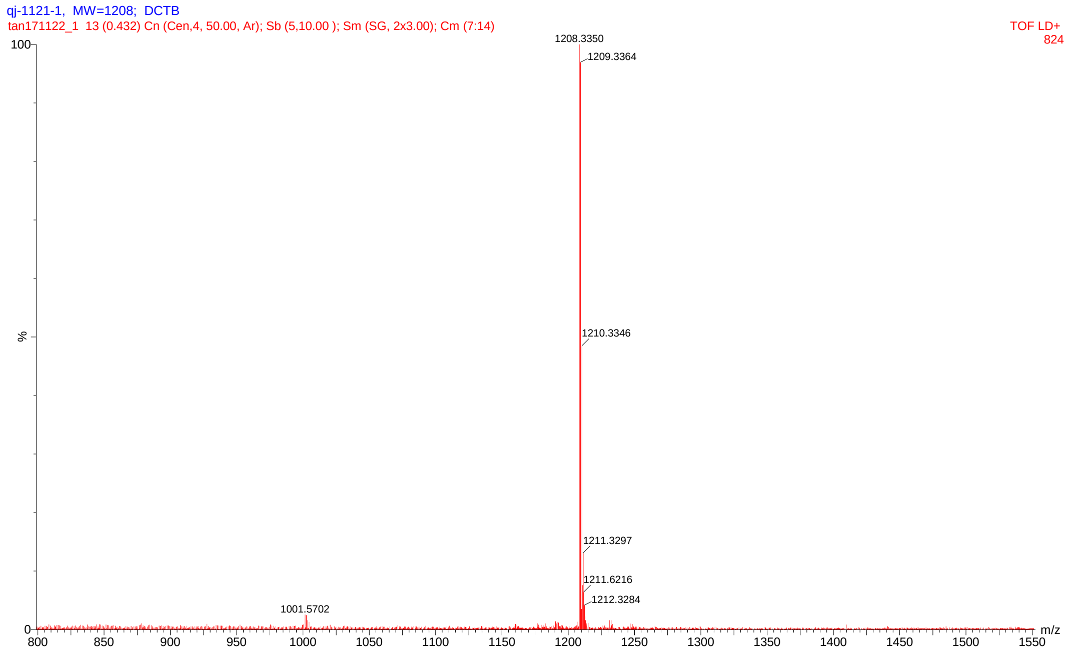

Supplementary Figure 17. HRMS of compound **RClosed-DTE-TPECM**.

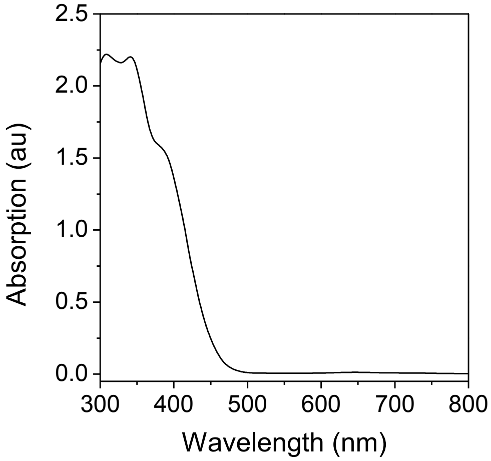

Supplementary Figure 18. The absorption spectrum of ROpen-DTE-TPECM in THF.

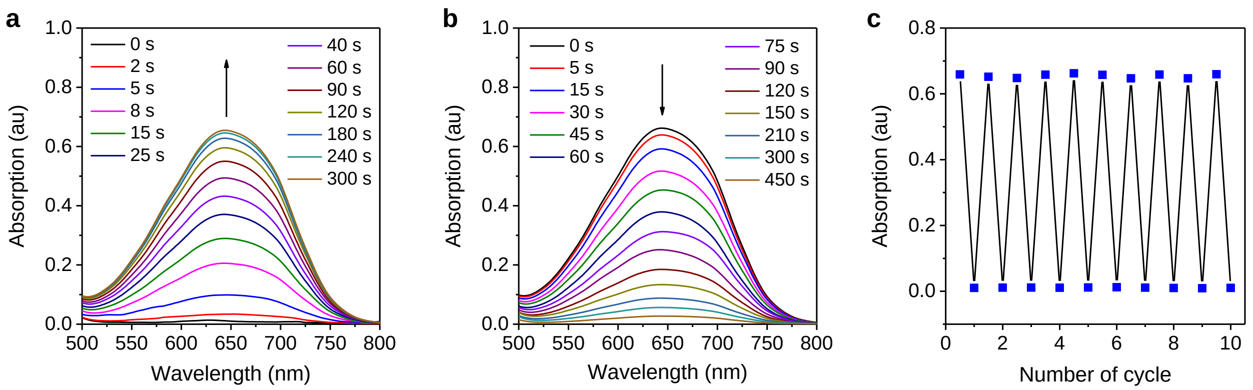

Supplementary Figure 19. Photoreversibility of DTE-TPECM in THF under UV/visible light irradiation. a,**b**, Absorption spectra of (**a**) ROpen-DTE-TPECM (THF solution) under UV light (365 nm, 0.1 W cm^{-2}) irradiation and (**b**) RClosed-DTE-TPECM (THF solution) under red light (610 nm, 0.3 W cm^{-2}) irradiation for different times. **c**, The absorption intensity at 650 nm of DTE-TPECM in THF during ten circles of red (610 nm, 0.3 W cm^{-2}, 10 min)-UV (365 nm, 0.1 W cm^{-2}, 5 min) light irradiation processes.

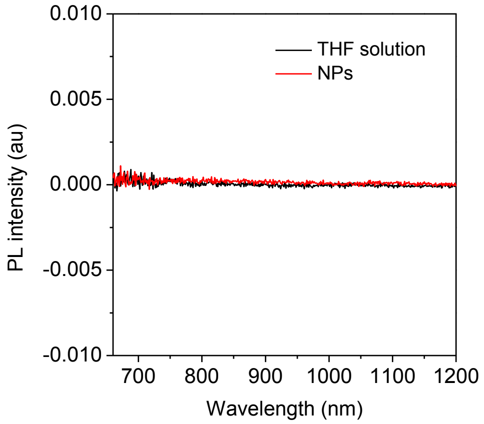

Supplementary Figure 20. The PL spectra of the RClosed-DTE-TPECM in THF and RClosed NPs in aqueous media.

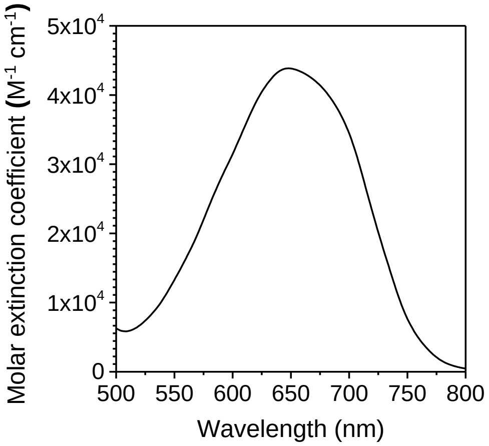

Supplementary Figure 21. Absorption spectrum of RClosed NPs (based on RClosed-DTE-TPECM) in aqueous solution.

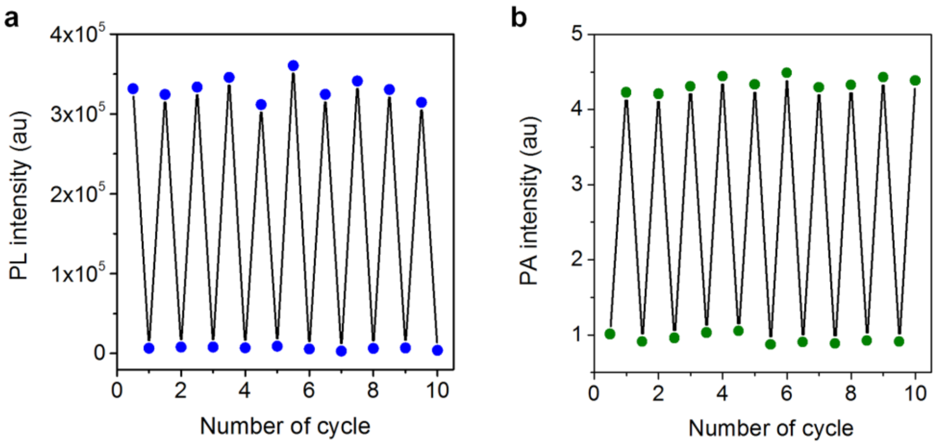

Supplementary Figure 22. Reversible property and bistable photochromism. a, The PL intensity at 550 nm and **b**, PA intensity at 700 nm of the NPs during ten circles of UV light (365 nm, 0.1 W cm^{-2})/visible (610 nm, 0.3 W cm^{-2}) irradiation processes.

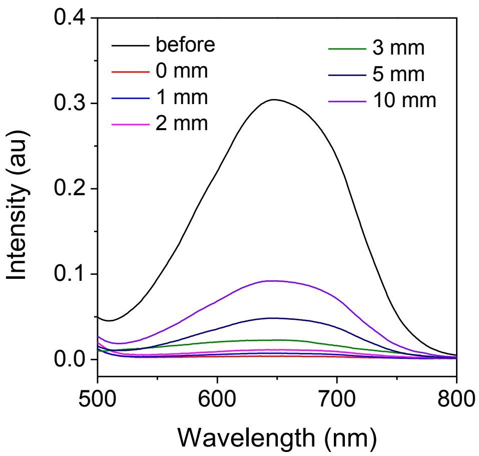

Supplementary Figure 23. The absorption spectra of the RClosed NPs before and after red light (610 nm, 0.3 W cm^{-2}) irradiation through different thicknesses of chicken breast as indicated.

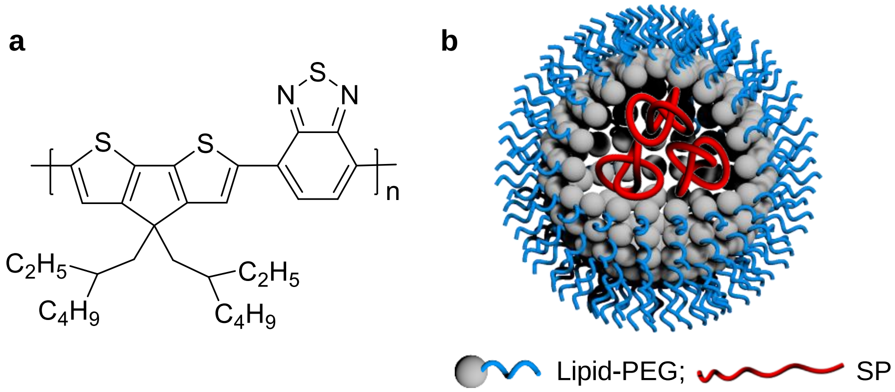

Supplementary Figure 24. Semiconducting polymer nanoparticle (SPN) used in this study. **a**, Chemical structure of SP: poly(cyclopentadithiophene-alt-benzothiadiazole)[7,8]. **b**, Schematic of the SPN with DSPE-PEG$_{2000}$ as the encapsulation matrix.

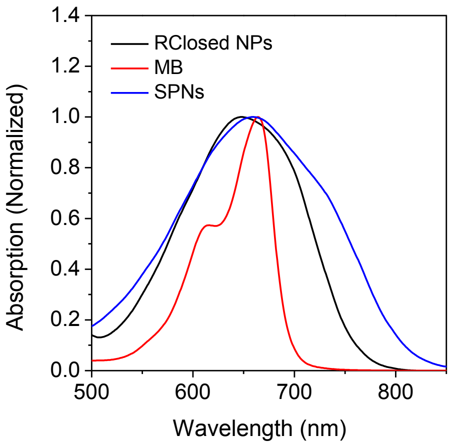

Supplementary Figure 25. The absorption spectra of RClosed NPs, SPNs and MB in water.

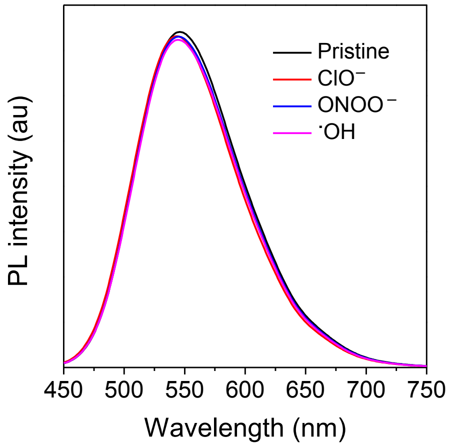

Supplementary Figure 26. PL spectra of the ROpen NPs with various RONS (400 μM) treatments as indicated.

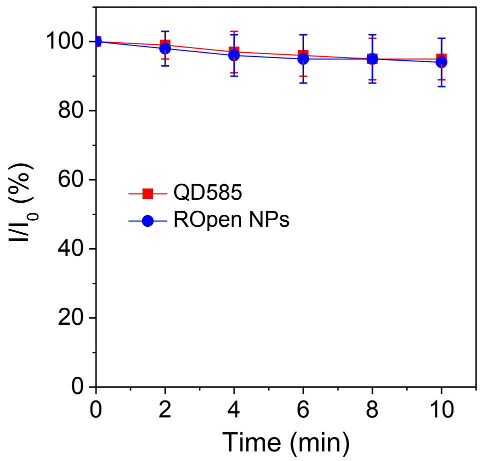

Supplementary Figure 27. Photostability comparison between ROpen NPs and QD585 in 4T1 cancer cells under continuous irradiation for 10 min. I_0 and I are the initial PL intensity and the PL intensity of each sample at different time points. Error bars, mean ± s.d. (n = 3).

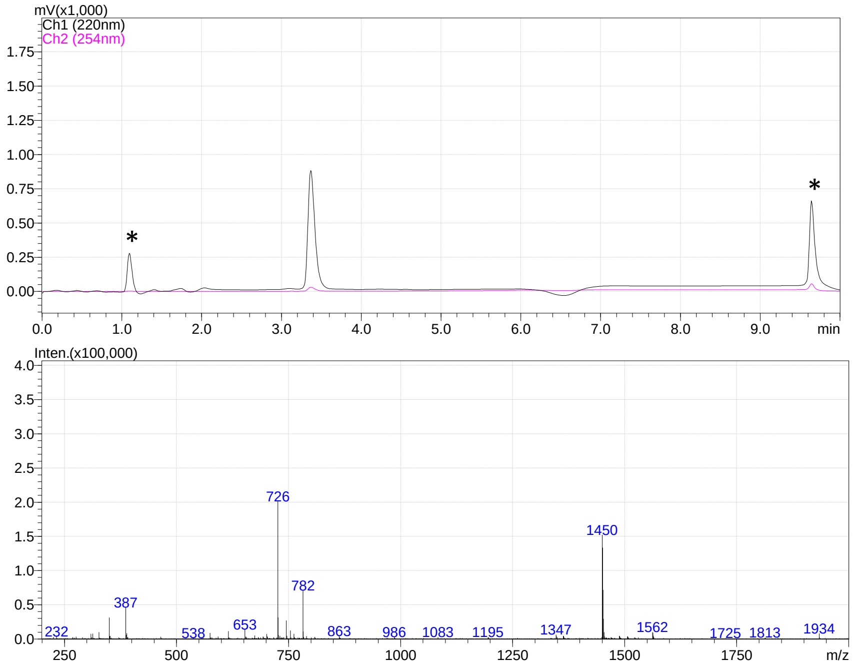

Supplementary Figure 28. LC-MS spectrum of CYSAYPDSVPMMS peptide (the stars represent systemic peaks).

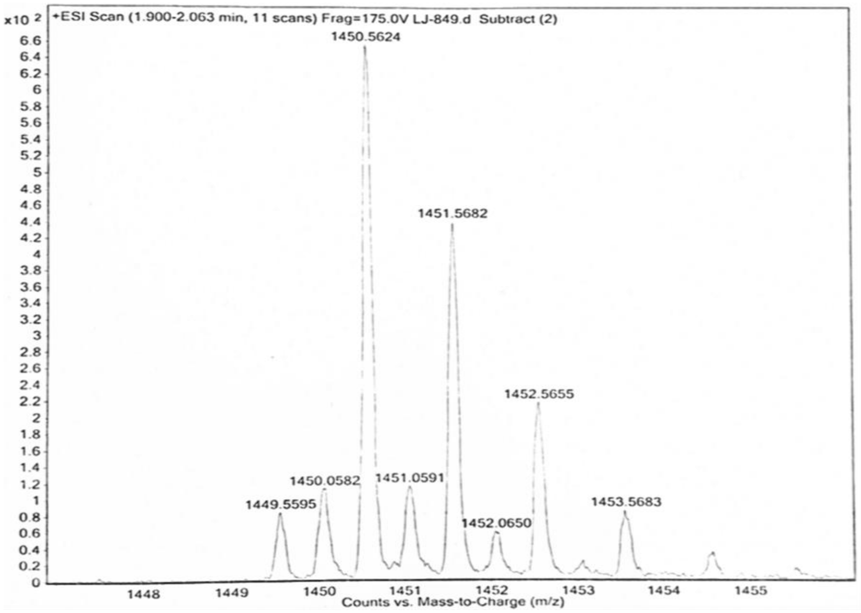

Supplementary Figure 29. HRMS spectrum of CYSAYPDSVPMMS peptide.

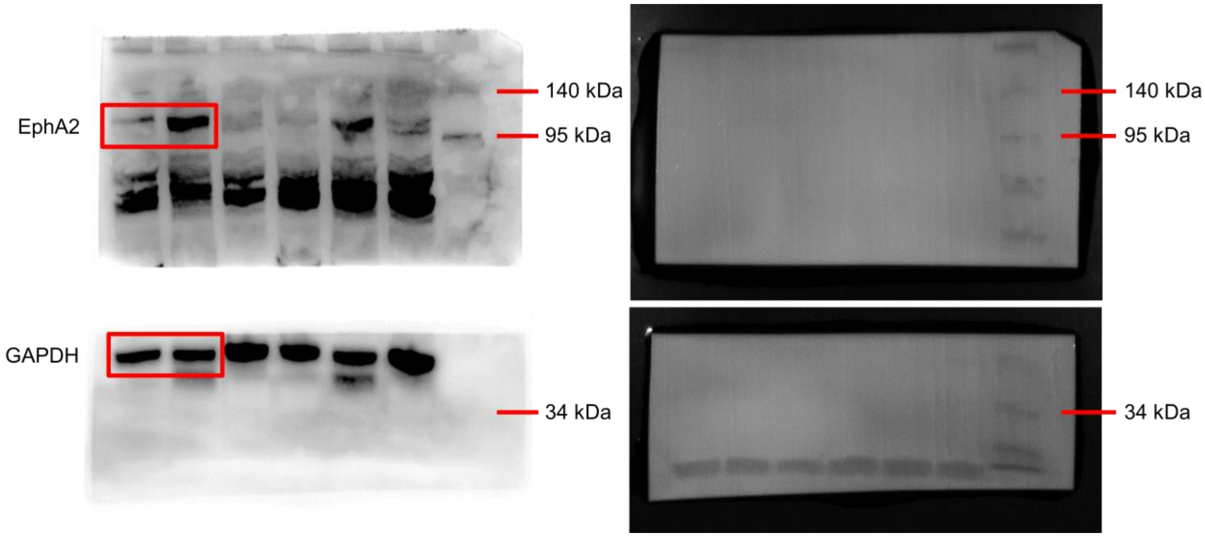

Supplementary Figure 30. Uncropped western blot scans used in Fig. 5c.

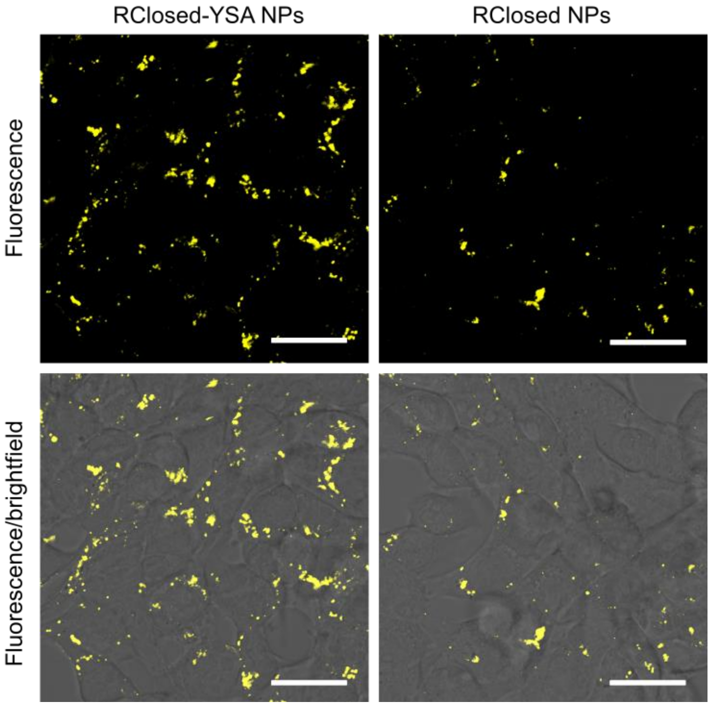

Supplementary Figure 31. CLSM fluorescence image and fluorescence/brightfield overlay image of live 4T1 cancer cells after incubation with ROpen-YSA NPs and ROpen NPs (both at 8 μM based on ROpen-DTE-TPECM) for 4 h, respectively. Scale bars, 20 μm.

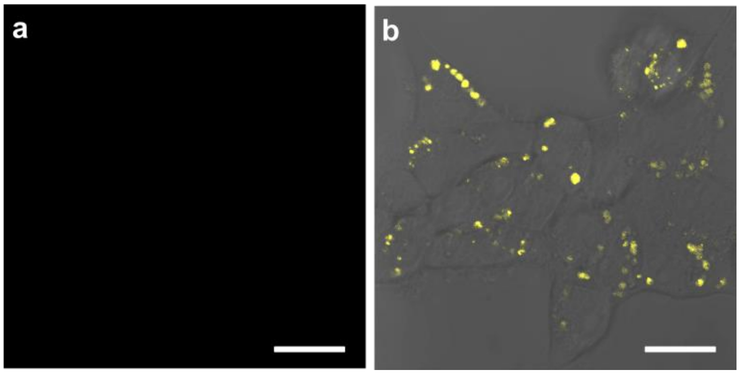

Supplementary Figure 32. Transformation of RClosed-YSA NPs to fluorescent ROpen-YSA NPs inside the cancer cells under red light irradiation. a, CLSM fluorescence image of RClosed-YSA NP-incubated 4T1 cancer cells before red light irradiation, indicating that there is negligible fluorescence within the cells. **b**, CLSM fluorescence/brightfield overlay image of RClosed-YSA NP-incubated 4T1 cancer cells after exposure to 610 nm red light (0.3 W cm^{-2}) for 5 min. Obvious fluorescence signal is observed inside the cells, suggesting that RClosed-YSA NPs successfully convert to emissive ROpen-YSA NPs within 4T1 cancer cells. Scale bars, 20 μm for (**a**) and (**b**).

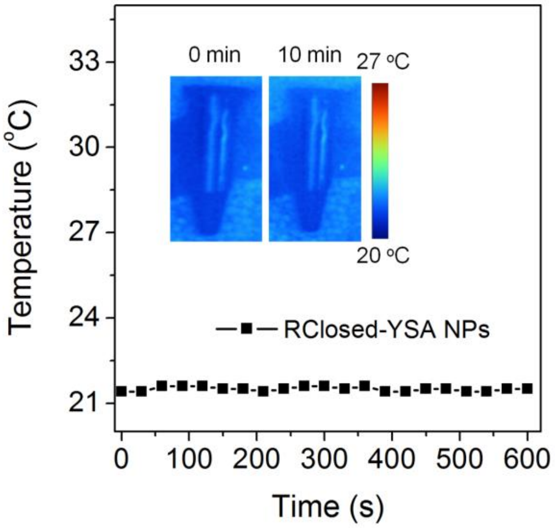

Supplementary Figure 33. The temperature of the RClosed-YSA NP suspension *versus* the 610 nm red light (0.3 W cm^{-2}) irradiating time. Inset shows the corresponding infrared (IR) thermal images before and after red light exposure for 10 min.

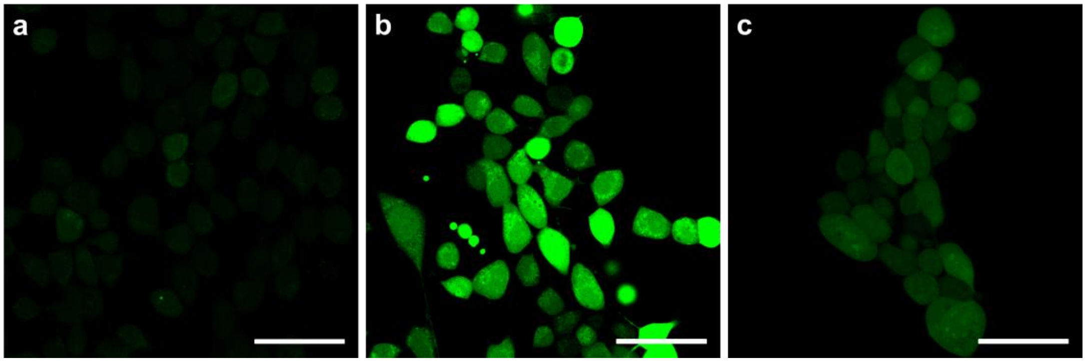

Supplementary Figure 34. Confirmation of ROS generation of transformed ROpen-YSA NPs inside the cancer cells. a-**c**, CLSM images show the intracellular ROS levels of 4T1 cancer cells treated with (**a**) transformed ROpen-YSA NPs only, (**b**) "transformed ROpen-YSA NPs + white light (0.25 W cm^{-2} for 2 min)", and (**c**) "transformed ROpen-YSA NPs + white light (0.25 W cm^{-2} for 2 min) + NaN_3 (10 mM)". DCF-DA was used as the ROS indicator. Scale bars, 50 μm for (**a-c**). As shown in Supplementary Fig. 34a, there is very low fluorescence of DCF inside the transformed ROpen-YSA NP-loaded cells without white light exposure. Nevertheless, upon irradiation by white light for 2 min, strong green fluorescence can be clearly observed within the transformed ROpen-YSA NP-loaded cells (Supplementary Fig. 34b), suggesting effective intracellular ROS production by "transformed ROpen-YSA NPs + white light". After the cells were co-treated with NaN_3, a singlet oxygen scavenger, the DCF fluorescence in the "transformed ROpen-YSA NPs + white light"-treated cells is significantly reduced (Supplementary Fig. 34c), revealing that the ROS generated by "transformed ROpen-YSA NPs + white light" is mainly singlet oxygen.

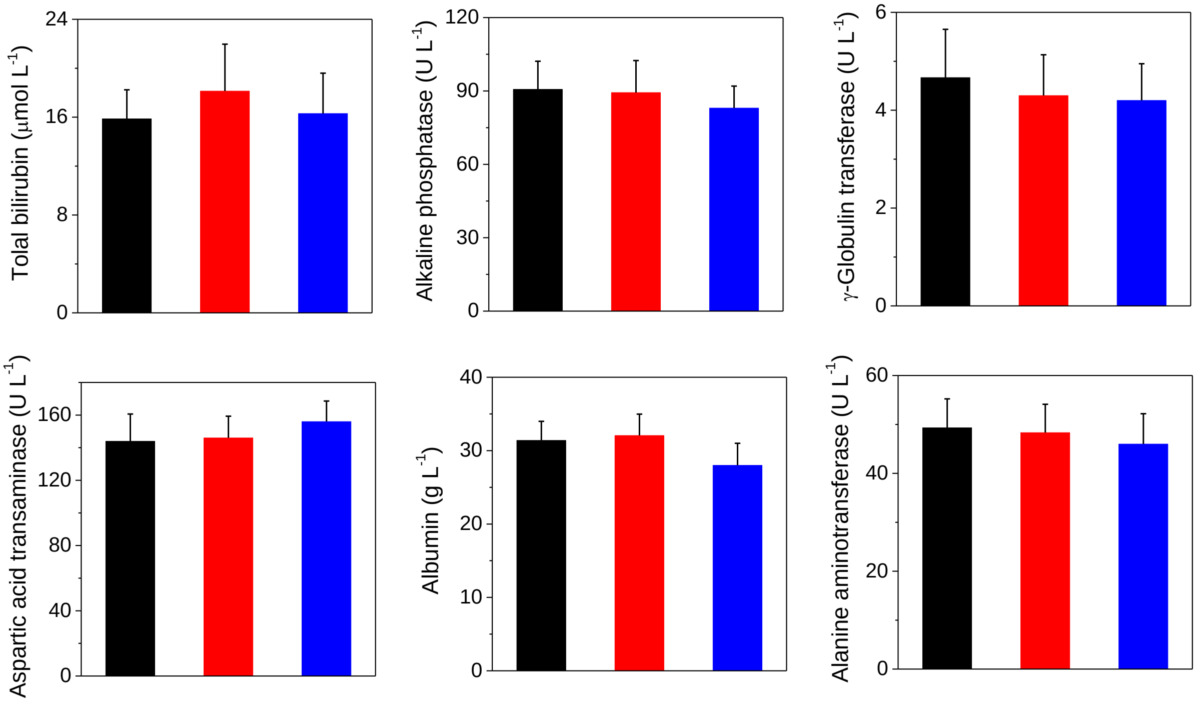

Supplementary Figure 35. Blood chemistry tests regarding to liver function of healthy mice after intravenous injection of RClosed-YSA NPs (red) and ROpen-YSA NPs (blue) for 7 days, respectively. The untreated mice (black) were utilized as a control. Error bars, mean ± s.d. (n = 4).

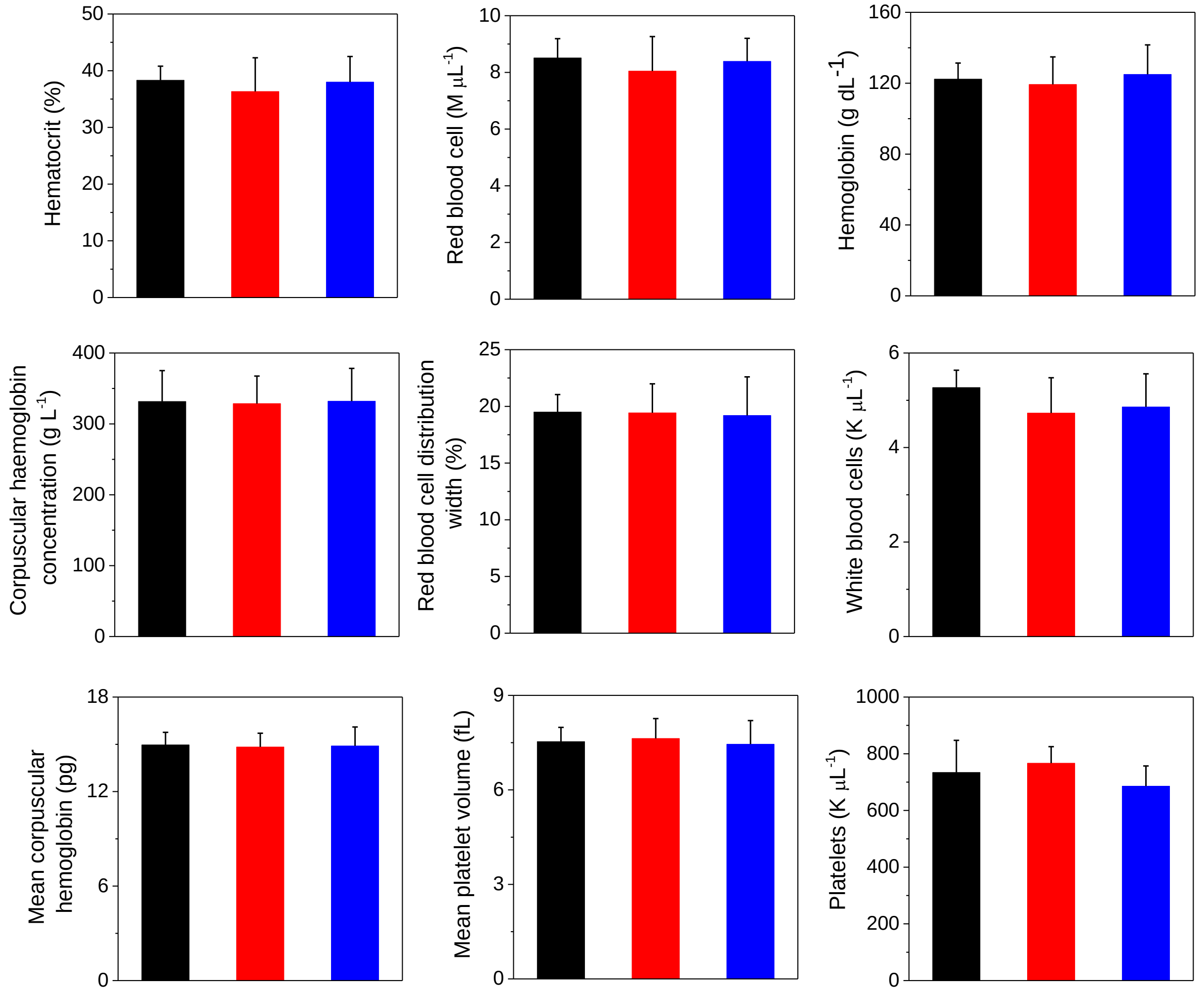

Supplementary Figure 36. Blood chemistry tests regarding to red blood cell, haem regulation and white blood cell count of healthy mice after intravenous injection of RClosed-YSA NPs (red) and ROpen-YSA NPs (blue) for 7 days, respectively. The untreated mice (black) were utilized as a control. Error bars, mean ± s.d. (n = 4).

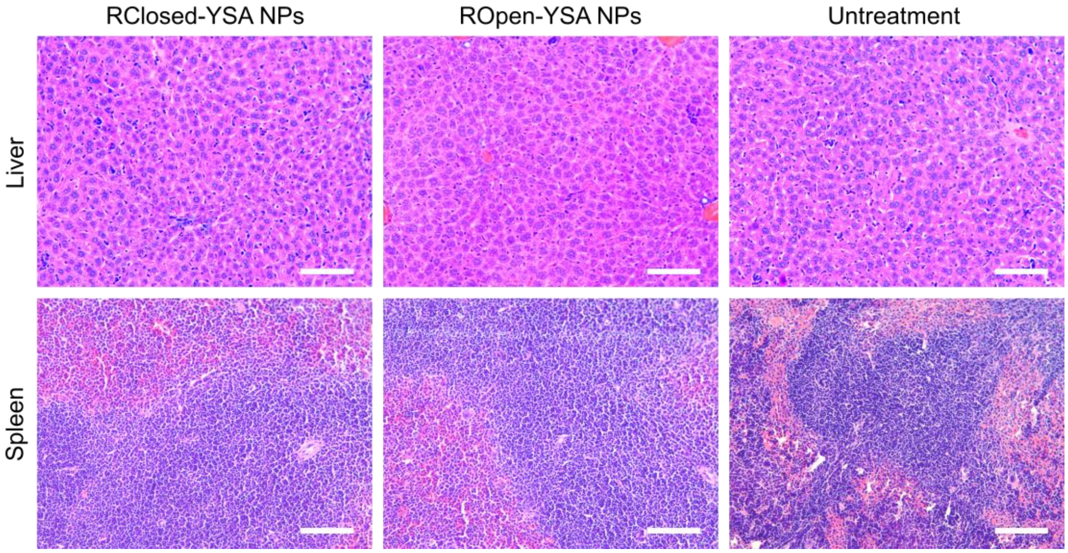

Supplementary Figure 37. Histological H&E stained images of liver and spleen on day 7 post various treatments as indicated. Scale bars, 100 μm.

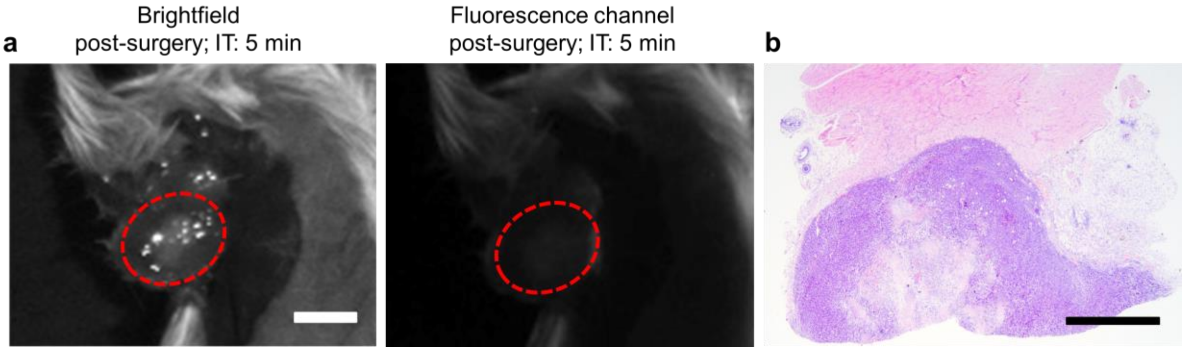

Supplementary Figure 38. *In vivo* imaging of saline-treated mice post-surgery. a, Representative brightfield image and fluorescence image of saline-treated tumour-bearing mice with residual tumours after resection, followed by 610 nm red light (0.3 W cm^{-2}) irradiation at the operative incision site for 5 min. The mice were not injected with any NPs. IT: irradiation time. The red dashed circles indicate the operative incision site with residual tumour. Scale bar, 4 mm. **b**, H&E stained tissues at the operative incision site in (**a**) indicate the existence of residual tumour. Scale bar, 1 mm.

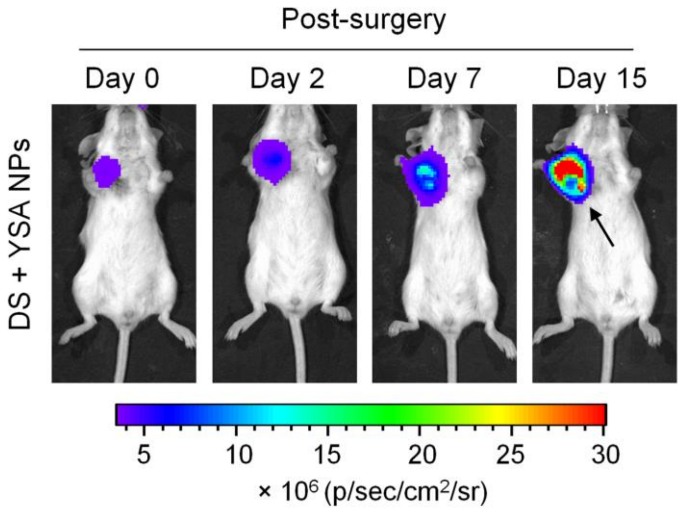

Supplementary Figure 39. Time-dependent bioluminescence imaging of residual tumours from mice in "DS + YSA NPs". The tumours were debulked on day 0. The black arrows indicate the residual tumours. DS: debulking surgery.

Supplementary Table 1. Cartesian coordinates of optimized ROpen-DTE-TPECM calculated by the DFT, B3LYP/6-31G(d), Gaussian 09 program.

atom	x	y	z
C	7.1205	-1.0762	-1.5541
C	8.2259	-0.3164	-1.5767
C	8.4519	0.3083	-0.4084
S	7.4262	-0.1276	0.5493
C	6.5939	-0.9691	-0.3206
C	6.6965	-1.8252	-2.5905
C	5.4601	-2.3107	-2.822
C	4.2744	-1.7333	-2.5371
C	4.0068	-0.4324	-2.3135
S	2.5702	-0.1912	-2.1337
C	2.0496	-1.5547	-2.2897
C	3.0912	-2.3676	-2.5362

C	0.7396	-1.8893	-2.2271
C	9.4522	1.1722	-0.1203
C	5.5697	-3.5841	-3.6478
C	7.0509	-3.8203	-3.6744
C	7.6641	-2.4585	-3.5755
F	4.8989	-4.6312	-3.1512
F	5.0678	-3.3309	-4.865
F	8.95	-2.4896	-3.205
F	7.5787	-1.7895	-4.7333
F	7.4566	-4.5086	-4.725
F	7.3955	-4.5167	-2.6022
C	5.4476	-1.7562	0.2709
C	4.935	0.7539	-2.4496
C	-0.2514	-0.9753	-2.1559
C	-1.5498	-1.3144	-2.1115
C	-1.9525	-2.5989	-2.0778
C	-0.9666	-3.5154	-2.1318
C	0.3272	-3.1724	-2.2401
C	9.722	1.6018	1.1289
C	10.7143	2.465	1.3982
C	11.4815	3.0284	0.4425
C	11.2135	2.5841	-0.8026
C	10.2666	1.6696	-1.0706
C	12.4111	3.9869	0.7139
C	12.3271	4.7344	1.854
C	13.4314	4.2427	-0.1502
C	11.1136	5.0772	2.3627
C	13.4694	5.1046	2.4956
C	-3.2562	-2.9578	-1.9471
C	-3.5415	-4.0625	-1.2015
C	-4.2458	-2.2203	-2.5171
C	-2.8148	-4.2803	-0.0718
C	-4.5306	-4.91	-1.5908
C	-4.0308	-1.4557	-3.6099
C	-5.0054	-0.7356	-4.1876
C	-6.247	-0.7581	-3.6854
C	-6.4946	-1.5104	-2.605
C	-5.5092	-2.2256	-2.0392
C	-2.5827	-5.4992	0.4504
C	-1.8478	-5.6855	1.5597

C	-1.2639	-4.6817	2.2491
C	-1.5097	-3.4656	1.7242
C	-2.2477	-3.2724	0.6194
C	-5.2292	-5.6631	-0.7137
C	-6.2113	-6.4929	-1.0992
C	-6.5335	-6.5977	-2.3951
C	-5.868	-5.8552	-3.2897
C	-4.8919	-5.0258	-2.8876
C	13.5514	6.2366	3.2285
C	14.6812	6.6149	3.8471
C	15.7861	5.8637	3.7514
C	15.7405	4.7366	3.0292
C	14.6028	4.3731	2.4163
C	13.9522	5.4838	-0.2729
C	14.9608	5.7626	-1.1136
C	15.4972	4.793	-1.866
C	15.0132	3.5486	-1.7585
C	14.0027	3.2869	-0.9144
C	10.0146	5.221	1.5981
C	8.801	5.48	2.1136
C	8.5591	5.6159	3.433
C	9.6756	5.5009	4.1845
C	10.8935	5.2436	3.6806
C	-0.5189	-4.9248	3.3623
C	0.2788	-4.0289	3.992
C	-0.5592	-6.3358	3.9534
C	7.3431	5.8362	4.0077
C	6.1512	5.8808	3.3642
C	7.3009	6.0344	5.5268
C	0.9738	-4.3682	5.0627
N	1.5909	-4.6605	5.9996
C	0.5466	-2.7864	3.639
N	0.8259	-1.6983	3.3505
C	5.0256	6.0898	4.0237
N	4.0343	6.2774	4.5951
C	5.9	5.714	2.0801
N	5.6341	5.5748	0.9596
H	8.8421	-0.2042	-2.4781
H	2.9948	-3.4424	-2.7248
H	5.256	-2.7041	-0.2768

H	4.5145	-1.1494	0.291
H	5.6506	-2.0747	1.3192
H	4.4386	1.6232	-2.9389
H	5.2882	1.1074	-1.4554
H	5.8075	0.5397	-3.1032
H	-0.0445	0.1082	-2.1359
H	-2.2789	-0.4908	-2.0163
H	-1.2005	-4.5946	-2.1292
H	1.0386	-4.0112	-2.2949
H	9.1547	1.2375	2.0025
H	10.8719	2.6896	2.4664
H	11.7211	3.0077	-1.6853
H	10.1313	1.4141	-2.1338
H	-3.0491	-1.4285	-4.1121
H	-4.7912	-0.1356	-5.0883
H	-7.0522	-0.1705	-4.1562
H	-7.511	-1.5315	-2.1765
H	-5.7813	-2.791	-1.1325
H	-2.9415	-6.4101	-0.059
H	-1.712	-6.7411	1.8447
H	-1.1662	-2.5388	2.1986
H	-2.4173	-2.2206	0.3303
H	-5.0624	-5.5883	0.3738
H	-6.769	-7.0777	-0.3479
H	-7.3393	-7.2768	-2.718
H	-6.1212	-5.9341	-4.3606
H	-4.3683	-4.4703	-3.6838
H	12.7015	6.9361	3.3049
H	14.71	7.5567	4.4209
H	16.7181	6.1704	4.254
H	16.6403	4.1036	2.9477
H	14.6337	3.4137	1.8729
H	13.5369	6.3408	0.2847
H	15.345	6.7936	-1.1956
H	16.3281	5.0143	-2.5562
H	15.4626	2.7356	-2.3537
H	13.6987	2.2293	-0.8337
H	10.0851	5.1458	0.4986
H	8.0156	5.5765	1.3564
H	9.6532	5.5636	5.2839

H	11.7022	5.0874	4.4157
H	-1.6051	-6.7048	4.0549
H	-0.1689	-6.4259	4.9884
H	0.0392	-7.042	3.3334
H	7.5894	5.0965	6.0549
H	6.3154	6.3077	5.9559
H	7.9603	6.8756	5.8403

Supplementary Table 2. Cartesian coordinates of optimized Closed-DTE-TPECM calculated by the DFT, B3LYP/6-31G(d), Gaussian 09 program.

atom	x	y	z
C	3.5903	1.7062	-2.8518
C	2.2587	1.5284	-2.8077
C	1.8257	0.2478	-2.7953
S	2.9628	-0.6793	-2.9558
C	4.3471	0.4381	-3.1609
C	4.2807	2.8332	-2.6022
C	5.6052	2.7941	-2.3465
C	6.2548	1.6293	-2.1741
C	5.4603	0.3465	-2.1221
S	6.8554	-0.7257	-2.4534
C	7.9549	0.1161	-1.9334
C	7.5593	1.4089	-1.931
C	9.1485	-0.3862	-1.541
C	0.5696	-0.1558	-2.4905
C	6.1324	4.1868	-2.0998
C	4.8762	4.8295	-1.5802
C	3.7562	4.2342	-2.3875
F	7.1693	4.2793	-1.2603
F	6.5087	4.7059	-3.2761
F	2.5576	4.3284	-1.8024
F	3.6583	4.8137	-3.5921
F	4.9102	6.1481	-1.6231
F	4.7203	4.4894	-0.3102
C	4.8186	0.451	-4.6258
C	4.9702	0.1245	-0.6738

C	9.4867	-1.6841	-1.693
C	10.6215	-2.2027	-1.1989
C	11.4781	-1.4627	-0.4694
C	11.1803	-0.1518	-0.3763
C	10.0711	0.3753	-0.9203
C	0.2454	-1.4363	-2.2191
C	-0.9598	-1.7948	-1.7453
C	-1.9556	-0.9187	-1.4929
C	-1.6828	0.3118	-1.9772
C	-0.4697	0.6945	-2.4071
C	-3.1534	-1.262	-0.935
C	-4.1401	-0.3808	-0.5963
C	-3.4046	-2.5812	-0.7486
C	-3.9267	0.8969	-0.1766
C	-5.3785	-0.8376	-0.3013
C	12.5127	-2.0296	0.2051
C	12.8005	-1.5553	1.452
C	13.1747	-3.088	-0.3335
C	11.7851	-1.0654	2.215
C	14.0736	-1.5693	1.9281
C	13.2692	-3.2554	-1.6709
C	13.9302	-4.2848	-2.2234
C	14.5267	-5.1964	-1.4441
C	14.4505	-5.0601	-0.1135
C	13.7881	-4.0231	0.4236
C	10.5199	-1.5178	2.1219
C	9.5025	-0.9875	2.8187
C	9.6403	0.0422	3.6817
C	10.9169	0.462	3.7925
C	11.9424	-0.0631	3.1001
C	14.3427	-1.6632	3.2489
C	15.596	-1.7129	3.727
C	16.6383	-1.6678	2.8871
C	16.4056	-1.574	1.5712
C	15.1462	-1.5287	1.1083
C	-6.4158	-0.641	-1.1373
C	-7.6368	-1.1122	-0.8387
C	-7.8374	-1.7897	0.3016
C	-6.8127	-1.9923	1.1429
C	-5.5938	-1.5186	0.8415

C	-4.4128	-3.2226	-1.3723
C	-4.6633	-4.5218	-1.1461
C	-3.9103	-5.2118	-0.2774
C	-2.9087	-4.591	0.3617
C	-2.6642	-3.2924	0.1267
C	-4.8794	1.8484	-0.1299
C	-4.6262	3.1164	0.2365
C	-3.4106	3.5589	0.6232
C	-2.5003	2.5696	0.7043
C	-2.7493	1.3015	0.3399
C	8.5618	0.5616	4.3337
C	8.5783	1.5804	5.2274
C	7.1896	-0.0487	4.0306
C	-3.1868	4.8702	0.9146
C	-1.9754	5.4339	1.1417
C	-4.3951	5.8095	0.9462
C	9.6089	2.277	5.6652
N	10.4858	2.9131	6.0797
C	-1.8577	6.7257	1.3916
N	-1.7466	7.8598	1.606
C	7.4683	2.0097	5.8014
N	6.4998	2.3908	6.3127
C	-0.7877	4.8616	1.0917
N	0.2727	4.3939	1.0423
H	1.5981	2.3824	-2.6174
H	8.2453	2.245	-1.747
H	5.5729	1.2486	-4.8103
H	5.2789	-0.52	-4.9163
H	3.9772	0.6386	-5.3317
H	4.4988	-0.8759	-0.5478
H	4.2239	0.8911	-0.3663
H	5.7963	0.1806	0.0717
H	8.8207	-2.4086	-2.1913
H	10.7512	-3.2941	-1.3022
H	11.8473	0.5354	0.1736
H	9.9152	1.4512	-0.7438
H	0.9817	-2.2537	-2.3074
H	-1.059	-2.872	-1.5419
H	-2.4552	1.0948	-2.0336
H	-0.3743	1.7513	-2.7045

H	12.8452	-2.5192	-2.3753
H	13.9975	-4.3757	-3.3208
H	15.0719	-6.0442	-1.8907
H	14.9271	-5.8117	0.5386
H	13.7357	-3.9991	1.5247
H	10.2714	-2.3769	1.4752
H	8.5315	-1.4702	2.6258
H	11.2072	1.2976	4.4383
H	12.9259	0.4219	3.2296
H	13.5383	-1.7677	3.9967
H	15.7717	-1.8141	4.8115
H	17.6702	-1.7144	3.2724
H	17.2556	-1.5307	0.8697
H	15.0319	-1.4233	0.0164
H	-6.2693	-0.1045	-2.0907
H	-8.4757	-0.95	-1.5358
H	-8.8389	-2.1809	0.5458
H	-6.973	-2.5484	2.0819
H	-4.7693	-1.6934	1.5543
H	-5.0397	-2.7021	-2.1153
H	-5.4865	-5.0261	-1.6795
H	-4.1157	-6.2782	-0.0867
H	-2.2953	-5.1468	1.0909
H	-1.8561	-2.807	0.7023
H	-5.9112	1.6471	-0.4583
H	-5.4916	3.7931	0.1522
H	-1.4935	2.7285	1.1075
H	-1.9314	0.58	0.5108
H	6.3266	0.4143	4.5505
H	6.9388	0.0571	2.9499
H	7.1526	-1.1191	4.3378
H	-5.1893	5.4206	1.6234
H	-4.803	5.9599	-0.0799
H	-4.2026	6.8288	1.3399

Supplementary Table 3. Pharmacokinetic data of ^{125}I-labelled RClosed-YSA NPs after intravenous injection (mean ± SD, *n* = 6 rats).

Parameters	^{125}I-labelled RClosed-YSA NPs	Unit
$t_{1/2}$: Half-life	6.21 ± 0.39	h
$AUC_{(0-\infty)}$: Area under the curve	205.31 ± 19.94	μg h mL^{-1}
CL: Total blood clearance	24.47 ± 2.38	mL kg^{-1} h^{-1}
Vd: Volume of distribution	143.03 ± 12.12	mL kg^{-1}
C_{max}: Maximum concentration	49.97 ± 3.91	μg mL^{-1}
MRT: Mean residence time	5.85 ± 0.07	h

References

1. Wang, W. *et al.* MicroRNA-592 targets IGF-1R to suppress cellular proliferation, migration and invasion in hepatocellular carcinoma. *Oncol. Lett.* **13**, 3522–3528 (2017).

2. Willms, E. *et al.* Cells release subpopulations of exosomes with distinct molecular and biological properties. *Sci. Rep.* **6**, 22519 (2016).

3. Fischer, H. C., Liu, L., Pang, K. S. & Chan, W. C. W. Pharmacokinetics of nanoscale quantum dots: *In vivo* distribution, sequestration, and clearance in the rat. *Adv. Funct. Mater.* **16**, 1299–1305 (2006).

4. Mori, K. *et al.* One-color reversible control of photochromic reactions in a diarylethene derivative: Three-photon cyclization and two-photon cycloreversion by a near-infrared femtosecond laser pulse at 1.28 μm. *J. Am. Chem. Soc.* **133**, 2621–2625 (2011).

5. Zhang, J., Zou, Q. & Tian, H. Photochromic materials: More than meets the eye. *Adv. Mater.* **25**, 378–399 (2013).

6. Chen, H. *et al.* Design of a photoactive hybrid bilayer dielectric for flexible nonvolatile organic memory transistors. *ACS Nano* **10**, 436–445 (2016).

7. Pu, K. *et al.* Diketopyrrolopyrrole-based semiconducting polymer nanoparticles for *in vivo* photoacoustic imaging. *Adv. Mater.* **27**, 5184−5190 (2015).

8. Pu, K. *et al.* Semiconducting polymer nanoparticles as photoacoustic molecular imaging probes in living mice. *Nat. Nanotechnol.* **9**, 233−239 (2014).
